# Vascular motion in the dorsal root ganglion sensed by Piezo2 in sensory neurons triggers episodic neuropathic pain

**DOI:** 10.1016/j.neuron.2025.03.006

**Published:** 2025-03-27

**Authors:** Wenrui Xie, Debora Denardin Lückemeyer, Katherine A. Qualls, Arthur Silveira Prudente, Temugin Berta, Mingxia Gu, Judith A. Strong, Xinzhong Dong, Jun-Ming Zhang

**Affiliations:** 1 Pain Research Center, Department of Anesthesiology, University of Cincinnati College of Medicine, Cincinnati OH 45267, USA; 2 The Solomon H. Snyder Department of Neuroscience, Johns Hopkins University School of Medicine, Baltimore, MD 21209, USA; 3 Howard Hughes Medical Institute, Johns Hopkins University School of Medicine, Baltimore, MD 21209, USA; 4 Department of Anesthesiology & Perioperative Medicine, David Geffen School of Medicine, University of California, Los Angeles, Los Angeles, CA 90095, USA; 5 Lead Contact

## Abstract

Spontaneous pain, characterized by episodic shooting or stabbing sensations, is a major complaint among neuropathic pain patients yet its mechanisms remain poorly understood. Recent research indicates a connection between this pain condition and “clustered firing”, wherein adjacent sensory neurons fire simultaneously. This study presents evidence that the triggers of spontaneous pain and clustered firing are the dynamic movements of small blood vessels within the nerve-injured sensory ganglion, along with increased blood vessel density/angiogenesis and increased number of pericytes around blood vessels. Pharmacologically or mechanically evoked myogenic vascular responses increase both spontaneous pain and clustered firing in a mouse model of neuropathic pain. The mechanoreceptor Piezo2 in sensory neurons plays a critical role in detecting blood vessel movements. An anti-VEGF monoclonal antibody that inhibits angiogenesis effectively blocks spontaneous pain and clustered firing. These findings suggest targeting Piezo2, angiogenesis, or abnormal vascular dynamics as potential therapeutic strategies for neuropathic spontaneous pain.

## eTOC

Spontaneous pain, characterized by episodic shooting or stabbing sensations, is a significant complaint among neuropathic pain patients but remains poorly understood. Xie et al. provide evidence that triggers of spontaneous pain and clustered firings are dynamic movements of blood vessels in sensory ganglion, detected by Piezo2 receptors in adjacent neurons.

## Graphical Abstract

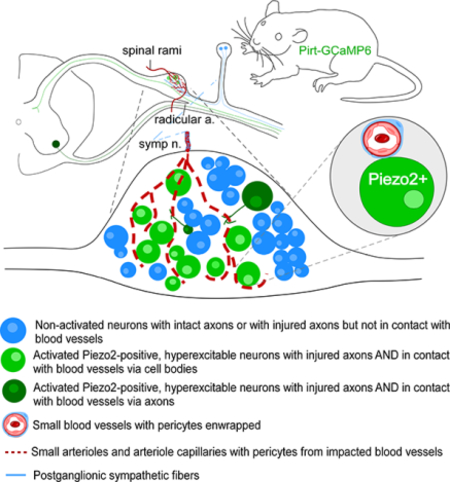

## Introduction

Intractable spontaneous pain, such as episodic shooting and stabbing pain that occurs without external stimuli, is a primary complaint among neuropathic pain patients^1^. Unlike evoked pain caused by increased neuronal sensitivity, the mechanisms for episodic spontaneous pain may differ in neuropathic pain patients and animal models^2^. Using in vivo Ca^2+^ imaging of lumbar dorsal root ganglia (DRG) we recently showed that neuropathic spontaneous pain correlated with a novel form of sensory neuron activity in which spatially clustered neurons spontaneously activated together sporadically^3^. However, the mechanistic underpinnings of this clustered firing and its associated spontaneous pain remain to be elucidated.

Observing the spatial pattern of clustered firing, we noticed that most clustered firing events occur in regions of the DRG with enriched sympathetic fibers and blood vessels^3^. The intact DRGs receive blood supply from spinal rami of the nearby arteries and the posterior radicular artery^4,5^. It was reported previously that DRGs with injured nerve exhibited increased vascular branching, irregularity, and tortuosity, and that neurons with injured axons are often enveloped by vascular loops^4^. This led us to postulate that blood vessel activity or movement nearby might trigger clustered neuronal firing.

Pericytes are contractile cells associated with capillaries, arterioles, and venules, playing central roles in regulating blood flow, vascular development/angiogenesis, blood-brain barrier maintenance, neuroprotection, and neuroinflammation^6–8^. They are characterized by their elongated shape and the presence of contractile proteins, enabling them to interact closely with endothelial cells of blood vessels.

Piezo2, a mechanoreceptor found in most sensory neurons but not in motor and sympathetic neurons^9^, detects mechanical forces and mediates touch, proprioception, lung stretch, bladder fullness, and baroreception^10^. Piezo2 is also expressed in nociceptive sensory neurons^9,11^ and plays a role in mechanical pain sensitivity and touch-induced pain after inflammation or nerve injury^12–18^. However, these studies primarily examined evoked mechanical pain rather than spontaneous pain. Unlike Piezo2, Piezo1 level in the DRG is very low and is selectively expressed in itch-specific neurons, which transduce mechanical itch in mice^19^.

In this study, we first examined altered vascularization of the DRGs with nerve injury, followed by the characterization of the clustered firing and spontaneous pain that we observed during systemic or local administration of vasoconstrictors. This initial observation was confirmed by finding that this clustered firing could also be triggered by other chemical and mechanical manipulation of the local vasculature, and it was reduced by pharmacological and genetic inhibition of the mechanoreceptors, including Piezo2, in the injured DRG, as well as by treatment with an anti-angiogenesis monoclonal antibody.

## Results

### Increased blood vessel density in the nerve injured DRGs

**Figure 1:**
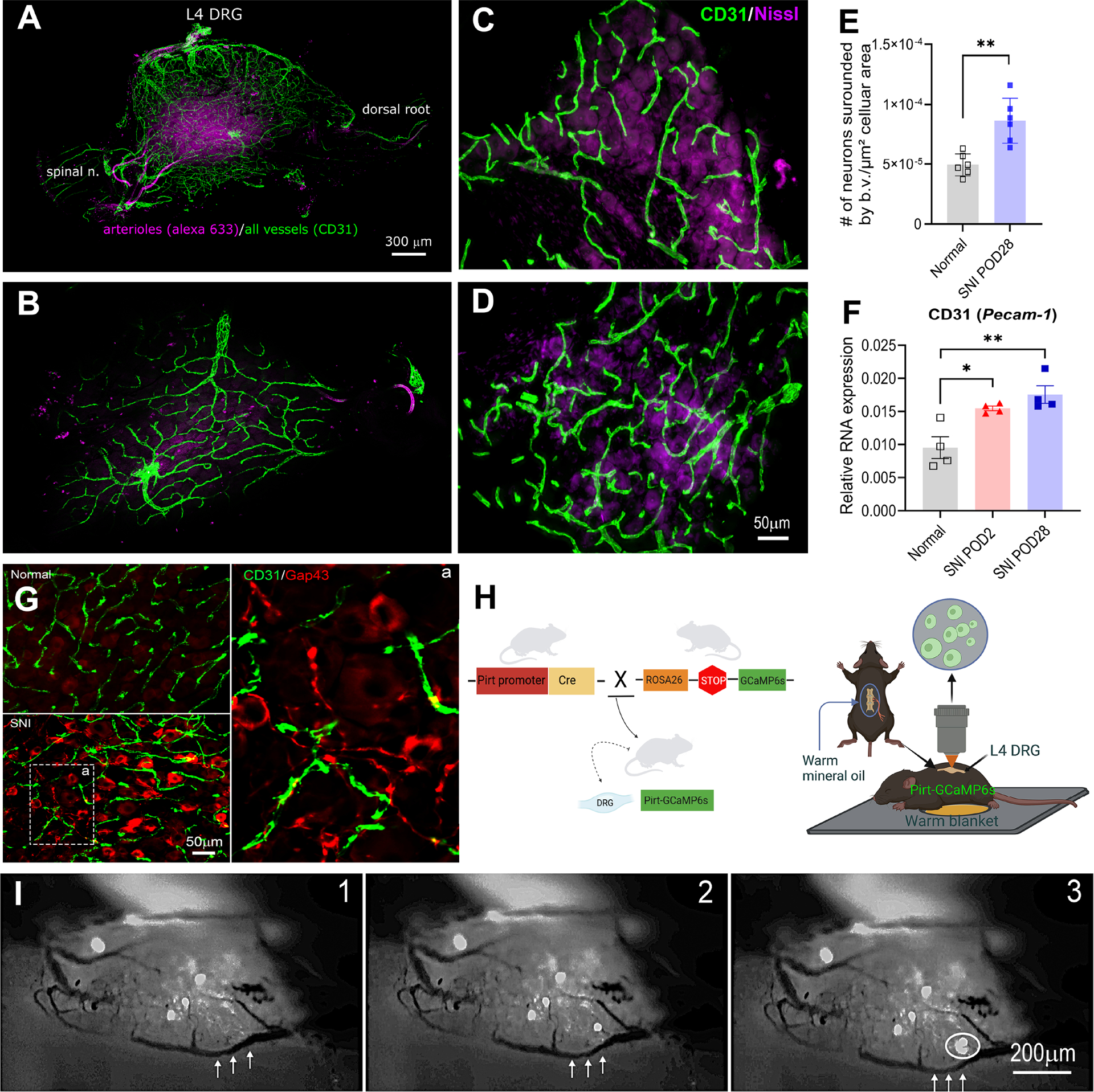
Vascularization of the DRG and vascular responses preceding clustered firing from DRG neurons of SNI mice **(A)** Projection of 3D volume imaging of a SNI L4 DRG labeled with antibody to pan endothelial cell marker CD31 (green) and Alexa 633 dye (far red; label for elastin in arterial structures). **(B)** Three large branches of blood vessels on the surface of the same DRG (top view). **(C, D)** Immunostaining of the blood vessels identified by CD31 in DRG sections from normal (C) and SNI (D) mice. **(E)** Summary data of C and D showing increased number of neurons in normal and SNI DRGs, with cell bodies surrounded more than half by CD31+ blood vessels. **, p<0.001, t-test, n=6 per group. **(F)** qPCR analysis indicated that CD31 mRNA expression increased in SNI mouse DRGs on POD 2 and POD 28 compared to normal, uninjured DRGs. *p<0.05, **p<0.01, ANOVA, n=4. **(G)** Labeling with GAP43 (red) revealed neuronal sprouting in the DRG after SNI, closely associated with blood vessels (CD31, green). **(H)** Diagram showing the mating strategy to express Ca indicator in sensory neurons and the in vivo recording setup used in (I). **(I)** Examples of single frames from video recordings showing vasomotion preceding clustered firing in SNI mice in vivo, with arrows indicating blood vessel movements. Blood vessel visibility was enhanced by i.v. dextran fluorescein (MW 70,000). See Movie S3 for dynamics.

Before conducting the live imaging experiments, we first examined the vascular distributions in mouse DRGs. We used a modified whole-DRG clearing technique combined with CD31 (Pecam1), a pan endothelial marker to label all blood vessels, including arterioles, venules and capillaries, along with Alexa 633 dye, a specific marker for smooth muscles to label elastin in arteriole structures. Confocal scanning of the whole mount DRG revealed an extremely high density of blood vessels (Figure 1A, side view of the DRG). Blood vessels originating from three large branches formed a network-like structure covering the entire surface of the DRG (Figure 1B, top view of the DRG, also see layer-by-layer scanning in Movie S1). Several small-diameter arteries entered the DRG from the spinal nerve-DRG junction, and another Alex 633+ artery could be seen entering the DRG from the top surface. In DRGs from mice with spared nerve injury (SNI), an established mouse model of neuropathic pain^20^, we observed increased blood vessel density or angiogenesis as demonstrated by the increased number of neurons encircled by blood vessels (Figure 1C–E) and increased expression of CD31 mRNA on POD2 and POD28 (Figure 1F). After nerve injury, increased blood vessel density enables greater interaction between vessels and neurons, as demonstrated by Gap-43-labeled axons appearing close to blood vessels in Figure 1G.

### Live microscopic observation of clustered firings associated with sporadic vascular movements

Using intravenous dextran, we were able to simultaneously visualize and record neuronal clustered firings and blood vessel dynamics in the same DRG using an in vivo calcium imaging setup (Figure 1H). Clustered firings from DRGs of SNI mice were recorded as we previously reported^3^. To identify neurons participating in the clustered firings, we injected tracer DiI in the paw after SNI, 5 days prior to recording to label uninjured axons. We found that labeled uninjured neurons were rarely contributors to clustered firing. Clustered firing occurred completely outside of the region where intact neurons are located or in regions where injured and uninjured neurons are mixed (Figure S1, extracted from Movie S2).

In reviewing the recorded calcium imaging data, we found evidence of sporadic and often abrupt vascular responses preceding these clustered firings in 17 of the 51 clustered firing events. These vascular events include sudden change in blood flow, small blood vessel displacement, and vasoconstriction or dilation (Figure 1I, extracted from Movie S3).

### Vasoconstrictors increase clustered firing and spontaneous pain behavior

The observation above led us to hypothesize that neuropathic spontaneous pain and clustered firing in the injured DRG may be due to the direct impact of the nearby small blood vessels on sensory neurons. We thus attempted to investigate the effects of vascular activity on spontaneous pain and clustered firing using pharmacological, genetic and mechanical stimulation of blood vessels in the nerve-injured DRGs.

**Figure 2:**
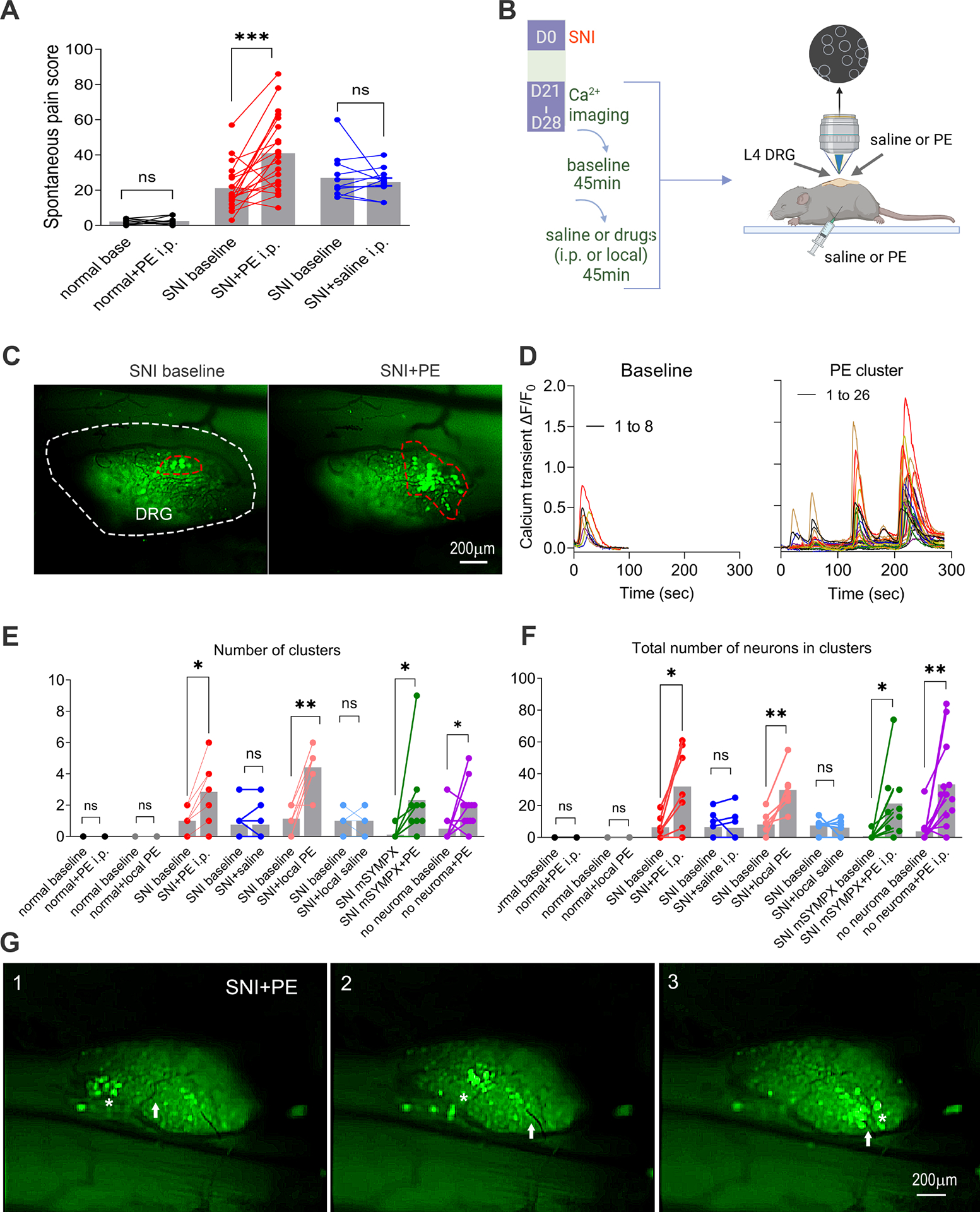
Vasoconstrictors enhance neuropathic spontaneous pain and abnormal clustered firing of the sensory neurons in SNI mice. **(A)** Systemic injection of phenylephrine (PE) increased spontaneous pain behaviors. Baseline pain was measured in sham animals (black) or after SNI was established (red, blue). PE (0.6 mg/kg, i.p) or vehicle (saline) was injected 2–3 hours later, and spontaneous pain was re-evaluated. Individual values are plotted as before-after points; bars indicate means. Black: in uninjured mice, baseline pain scores were low and unaffected by PE (p =0.52, paired t-test, n = 8. Red: after SNI, baseline pain increased and i.p. PE further increased spontaneous pain. ***, p<0.001, paired t-test, n = 21. Blue: Vehicle injection failed to show significant effects on baseline pain in SNI mice. p = 0.84., paired t-test, n =11. Conclusions were the same when analyzed for males only or females only. **(B)** Timelines of drug administration, and image recording setup. **(C)** Representative images (extracted frames from recordings) of the clustered firing before and after local PE application. **(D)** Time courses of calcium transients in individual neurons from the clusters before and after PE application. **(E, F)** Effect of PE on clustered firing. The number of clusters (E) and total number of neurons in clusters (F) was measured during 45 minutes of in vivo imaging (baseline). Then PE (0.6 mg/kg, i.p. or 0.3 mg/ml, 30 μl intra-DRG, “local”) or vehicle (buffered saline) was injected, and clusters observed for the next 45 minutes. In normal mice, no clusters were observed before or after i.p. PE (n = 5; black) or intra-DRG PE (n= 5; grey). In mice with established SNI (21–35 days), the number of clusters and total number of neurons per cluster were significantly increased by i.p. PE (n = 7; red) or local PE (n = 7; orange). In mice with established SNI, clustered firing was not affected by vehicle (saline) injection using the same recording protocols (n = 8 i.p., blue, and n = 7, local injection, light blue). Increase in clustered firing could also be observed in mice with microsympathectomy (“mSYMPX”) performed just prior to recording (n = 9; green). Clustered firing could still be induced in SNI mice in which the neuroma was disconnected from the DRG just prior to recording (n= 12; purple). **, p<0.01; *, p<0.05; n.s., not significant, paired t-test). **(G)** Example of single frames extracted from video recordings showing vasomotion preceding clustered firing in SNI mice in vivo. Clusters occurred at branches of blood vessels indicated by “*” in SNI DRG after PE (i.p.) and showing dynamic changes/displacement of small blood vessel indicated by the white arrow. Time between vascular activity and appearance of clustered firing is 4–5 sec.

We examined the effects of the α1 adrenergic receptor agonist and vasoconstrictor, phenylephrine (PE) in SNI mice, anticipating that local or systemic administration of PE would cause vasoconstriction in the DRG. We found that intraperitoneal (i.p.) injection of PE but not vehicle increased the spontaneous pain scores in SNI mice (Figure 2A). In contrast, sham control mice had low spontaneous pain scores that were not affected by PE. In vivo calcium imaging of the nerve injured DRGs showed that, in SNI mice, systemic injection of PE but not vehicle increased cluster incidence and number of neurons found in clusters (Figure 2B–G and Movie S4). While clusters occurred throughout the baseline recording period (45 minutes), clusters induced by PE were mainly concentrated in the first 10 – 15 minutes after injection (average delay = 7.3 ± 1.5 minutes), consistent with the previously reported ~10 minute time course for vasoconstriction to plateau after i.p. injection of PE in mice^21^.

Spontaneous clusters observed prior to PE injection were (in 4 out of 5 animals) enhanced by PE. However, PE often increased the numbers of neurons in these clusters and activated additional clusters. It also activated clusters in animals in which no clustered firing was observed prior to PE (n = 2 animals).

Clustered firing was never observed in sham control mice without peripheral nerve injury, even after PE injection (Figure 2E, 2F). Clusters evoked by PE also occurred outside of the region where intact neurons are located or mixed with neurons with intact axons. In 5 animals with PE – induced clusters containing a total of 360 neurons, only 4 cluster-firing neurons were also labeled by DiI, indicating that almost all neurons in cluster-firing are injured.

Clustered firing could also be enhanced by intra-DRG injection of PE, suggesting a local site of action (Figure 2E, 2F). As expected, this response was more rapid than the response to i.p. PE (average delay =1.56 ± 0.35 minutes). No increase was observed after intra-DRG injection of vehicle (Figure 2E, 2F). However, in all animals receiving intra-DRG vehicle injections, enhanced clustered firing remained visible after i.p. PE injection, confirming that the local drug application method did not interfere with the phenomenon (p<0.01 for both number of clusters and number of neurons in clusters; data not shown). Supporting a local DRG action, PE-induced clustered firing persisted even when the neuroma was disconnected from the DRG by cutting the nerve proximal to the neuroma before starting the recording session (Figure 2E, 2F, purple).

Similar to the baseline recordings, we found that clustered firing evoked by PE often occurs at or near branches of larger blood vessels (Figure 2G, extracted from Movie S5). We also observed evidence of microvascular responses preceding PE-evoked clustered firing events. Out of 58 analyzed clustered firing events, abnormal vascular responses were detected prior to firing in 24 cases (Figure 2G, also Movie S5, S6, S7). These vascular responses included abrupt changes in blood flow, vasoconstriction, and small blood vessel displacement. This consistent pattern suggests a strong correlation between microvascular dynamics and the initiation of clustered firing events in the DRG following PE administration. These findings underscore the significant role of vascular responses in modulating neuronal activity in neuropathic pain conditions.

We hypothesized earlier that the previously observed dependence of spontaneous clustered firing on local sympathetic fibers^3^ might reflect in part vasoconstriction events induced by spontaneous sympathetic discharges. Consistent with this idea, acute local microsympathectomy (mSYMPX) just prior to in viv*o* image recording strongly reduced baseline clustered firing (incidence and number of neurons in clusters) to about 11% (P<0.05), but i.p. PE could still significantly increase clustered firing (Figure 2E, 2F; green).

**Figure 3.**
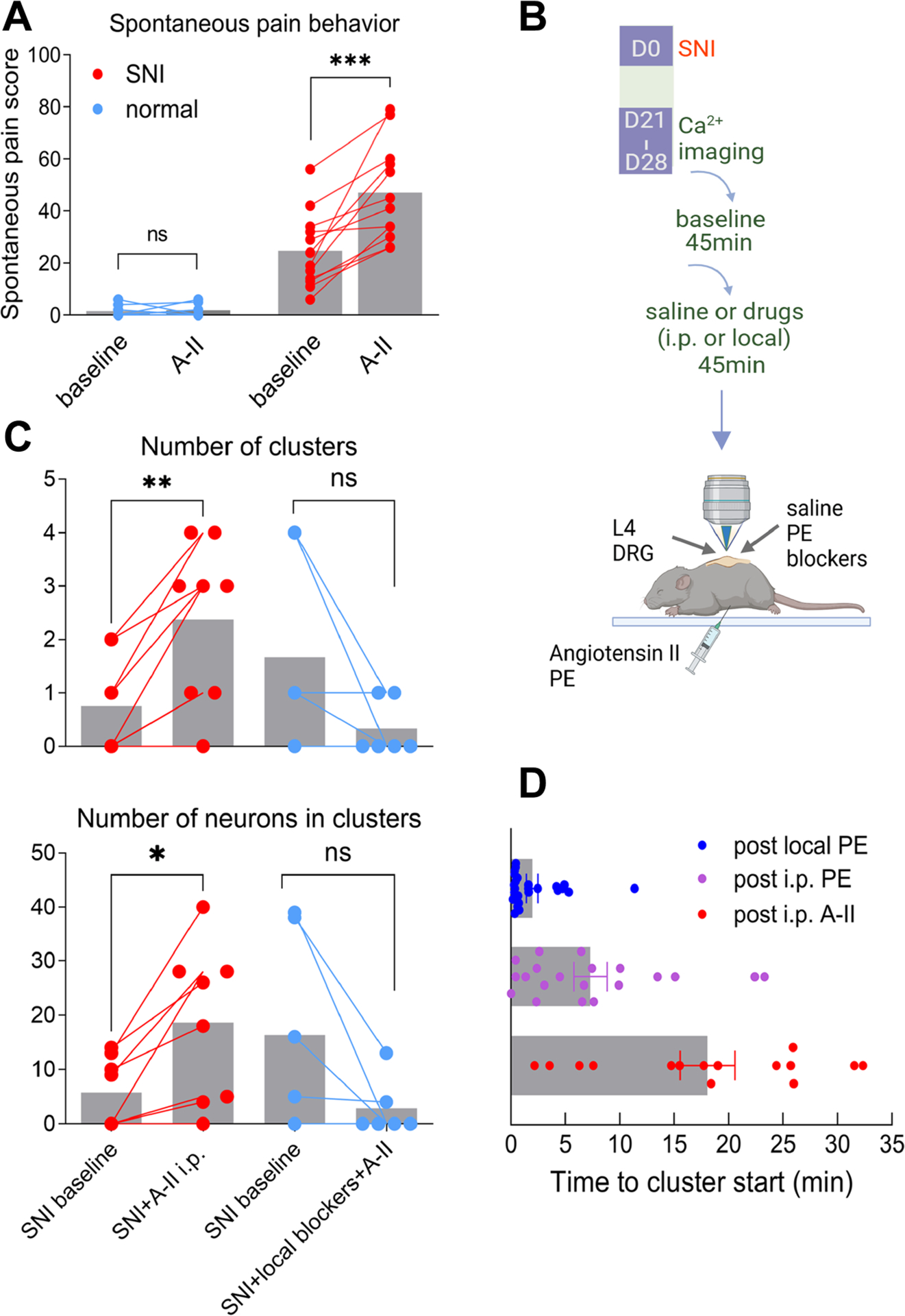
Angiotensin II (A-II) also increases spontaneous pain and clustered firing in SNI mice-an effect reduced by mechanical receptor blockers. **(A)** Baseline spontaneous pain was measured in normal animals or after SNI was established. Angiotensin II (A-II; 0.2 mg/kg, i.p.) was injected 3–4 hours later and spontaneous pain measured again. n.s., not significant; ***, p<0.001, paired t test. Normal, n = 8; SNI, n = 12. Conclusions were the same when analyzed for males only or females only. **(B)** Timelines of drug administration and image recording. **(C)** Effect of A-II on clustered firing. The number of clusters (top) and total number of neurons in clusters (bottom) were measured during 45 minutes of in vivo imaging (baseline). Then angiotensin II (0.6 mg/kg, i.p) was injected and clusters observed for the next 45 minutes. *, p<0.05, **, p<0.01, paired t test. N = 8 mice for i.p. In a second experiment, D-GsMTx4 and gsmtx4, blockers of mechanoreceptors, were injected locally into the DRG prior to the A-II injection, which blocked A-II induced clustered firing (n.s., not significant; n = 6). **(D)** Time to cluster start following i.p. injections of PE and A-II.

PE is an α1 agonist while increased adrenergic sensitivity after nerve injury is mediated primarily by α2 adrenoceptors^22–25^. In addition, direct activation of sensory neurons by PE would not explain the clustered nature of the firing. We tested our vasomotor hypothesis with angiotensin II, a second vasoconstrictor, to further rule out direct activation of sensory neurons as a cause of PE-induced clustered firing. As we expected, angiotensin II increased spontaneous pain behavior (Figure 3A) and clustered firing (Figure 3B–3C) in SNI mice but not in control mice, similar to PE’s effects. The onset of angiotensin II-induced clustered firing was slower than PE induced clustered firing (Figure 3D), consistent with the slower effects on spontaneous pain that we observed with behavior testing.

To find out if clustered firing, spontaneous or evoked by vasoconstrictors, may result from mechanical forces on neurons due to vascular movements i.e. vasoconstriction, we next examined effects of mechanical channel blockers on cluster-firing generation. The angiotensin II-induced clustered firing was blocked by injecting D-GsMTx4 and gsmtx4, blockers of mechanically sensitive channels, into the DRG before the i.p. injection of angiotensin II (Figure 3C).

### Pharmacological and genetic blocking of Piezo2 in the DRG neurons reduces spontaneous pain and mitigates the effects of vasoconstrictors on clustered firing

**Figure 4.**
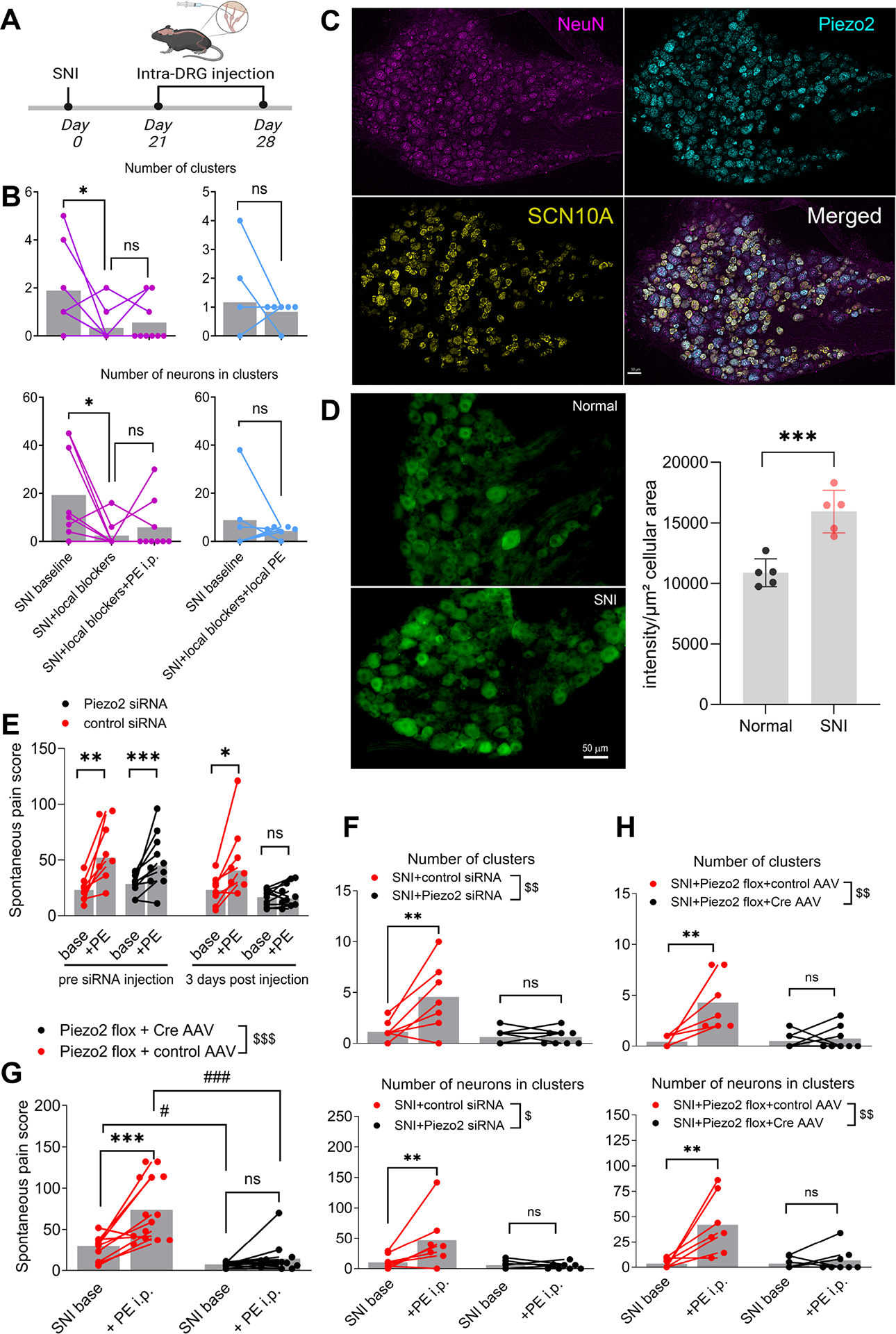
Role of mechanoreceptors in clustered firing and neuropathic spontaneous pain. **(A)** Timelines of local drug administration. **(B)** Effect of local mechanoreceptor blockers on clustered firing. Purple: After SNI was established, the L4 DRG was imaged for 45 minutes (“baseline”). D-GsMTx4 and gsmtx4 were injected into the DRG (2 μL, both at 50 μM) and the DRG imaged for another 45 minutes. Then blockers were re-injected, and PE given i.p. *, p<0.05; n.s., not significant, paired t-test (n=9). Blue: similar experiment format, but both PE and the blockers were injected together into the DRG (n=6, note, control for this experiment is intra DRG saline, shown in Figure 2E, F). **(C)** In situ hybridization confirming that some Na_V_1.8-positive neurons as putative nociceptors also express Piezo2 in L4 DRG from SNI mice on POD 28. Scale bar=50 μm. **(D)** Immunostaining demonstrating increased expression of Piezo2 in the SNI DRG on POD 28 compared to contralateral normal DRG (***, p<0.001, t-test, n = 5 per group). **(E)** Piezo2 knockdown with siRNA in the DRG blocks PE-induced increase in spontaneous pain. After SNI was established, mice were videotaped for 3 continuous days. On the third day, 1–2 hours after baseline taping (“base”), the mice received PE (i.p) and spontaneous pain was measured again (“+PE”). siRNA (against Piezo2, black, or control non-targeting, red) was injected two days later (i.t. 5 μl x2), and on the third day after siRNA injection, spontaneous pain before and after PE i.p. injection was measured again. The effect of PE on spontaneous pain was lost after Piezo2 knockdown. n.s., not significant; *, p<0.05,**, p<0.01, ***, p<0.001, significant difference between the indicated groups, two-way ANOVAs with Šídák’s posttest (n=8 per group). 2-way ANOVA of the difference scores (PE – baseline) showed a significant effect of siRNA type (p = 0.02) and of time (pre vs. post siRNA injection, p = 0.02). After Piezo2 siRNA injection the PE-induced change in pain behavior was not significantly different from zero, while all other difference scores were significantly different from zero (p<0.01 to 0.001.) **(F)** Effect of Piezo2 knockdown on baseline and PE-induced clustered firing. siRNA directed against Piezo2 or nontargeting control was injected after SNI was established, 2 – 3 days prior to recording. After recording baseline clustered firing, PE was injected i.p. N = 7 for control siRNA group, n=8 for siRNA group. $, p<0.05, $$, p<0.01, significant overall effect of siRNA, **, p<0.01, n.s., not significant effect of PE, two-way ANOVAs with Šídák’s posttest. **(G)** Effect of Cre-mediated Piezo2 knockdown on spontaneous pain behaviors. AAV-Cre or AAV-control virus was injected into the ipsilateral paw at age postnatal day 16. After the animals reached adulthood, SNI was performed. Spontaneous pain was measured 4–5 weeks later before (“SNI base”) and 1–2 hours after i.p. injection of PE. Piezo2 knockdown reduced spontaneous pain (#, p<0.05, ###, p<0.001 compared to AAV-Cre group) and blocked the PE-induced increase in spontaneous pain behaviors ($$$, p<0.001, significant overall effect of the AAV factor in 2-way RM ANOVA; ### or ***, p<0.001; # p<0.05, n.s., not significant, Šídák’s posttest). Cre group: n = 13; Control AAV group: n=14. **(H)** Effect of CRE-mediated Piezo2 knockdown on baseline and PE-induced clustered firing. Virus treatment was as in (G). Cre group: n=8; RFP control group (n=7). $$, p<0.01, significant overall effect of Cre factor, **, p<0.01, n.s., not significant effect of PE, 2-way ANOVAs with Šídák’s posttest.

To further examine the role in clustered firing of mechanically activated channels that might be detecting blood vessel movements, we examined the effects of D-GsMTx4 and gsmtx4 on PE-induced clustered firing. As shown in Figure 4A–B, local DRG injections of these blockers also reduced PE-induced clustered firing, similar to the findings with angiotensin II. As these nonspecific blockers affect several different mechanically activated channels, we next examined Piezo2 as a candidate mechanical receptor. Piezo2 was highly expressed in the DRG neurons (Figure S2). Using *in situ* hybridization, we confirmed that some putative nociceptors, defined as expressing the Na_V_1.8 sodium channel, coexpressed Piezo2 in DRG after SNI (Figure 4C), consistent with the previously observed relationship between spontaneous pain and clustered firing. In the L4 DRG after SNI, 45.2 ± 4.1% of neurons expressed both markers; 37.9 ± 2.8% Piezo2 only; 14.6 ± 2.0% Na_V_1.8 only; 2.3 ± 0.9% neither, (n=955 neurons in 3 animals). Similar co-expression was observed in uninjured contralateral DRG (55.1 ± 2.1% with both, 31.6 ± 1.1% Piezo2 only, 11.8 ± 1.4% % NaV1.8 only, and 1.5 ± 0.2% neither, n = 3/group, n.s. by paired t-tests). Immunohistochemical analysis showed that the expression of Piezo2 increased by 46% on POD28 in SNI mice, compared to sham control mice (Figure 4D).

To examine the possible role of Piezo2 more specifically, we knocked down Piezo2 in the lumbar DRG by intrathecal (i.t.) injection of siRNA. This blocked the increase in spontaneous pain behaviors induced by i.p. PE injection (Figure 4E) as well as PE-induced clustered firing (Figure 4F). In addition, comparing only the pain scores before PE injection showed that baseline spontaneous pain was also reduced by Piezo2 knockdown (to 59% of pre-siRNA value, p = 0.005, paired t-test) but not by control siRNA (100% of pre-siRNA value, p>0.99). The Piezo2 knockdown in the DRG was confirmed by IHC (Figure S3 top). For the time point examined (2 days after siRNA injection), Piezo2 signal in the neuroma was not affected (Figure S3 bottom), possibly reflecting the slow turnover and axonal transport of ion channels in distal axons. This provides further evidence for the hypothesis that the DRG was the main site of action for the PE-induced increases in spontaneous pain and clustered firing since any contribution from the neuroma would not have been affected by the siRNA.

To knockout Piezo2 in DRG neurons, mice with a floxed Piezo2 were injected in the ipsilateral hindpaw at postnatal day 16 with AAV expressing either Cre recombinase or control AAV expressing a red fluorophore. Virus expression and knockdown of Piezo2 in the DRG was confirmed with PCR and IHC (Figure S4). These mice also showed reduced baseline spontaneous pain scores and blocked their PE-induced increases in clustered firing in SNI mice (Figure 4G–H).

### Clustered firing can also be evoked by mechanically induced myogenic vascular responses in vivo

**Figure 5.**
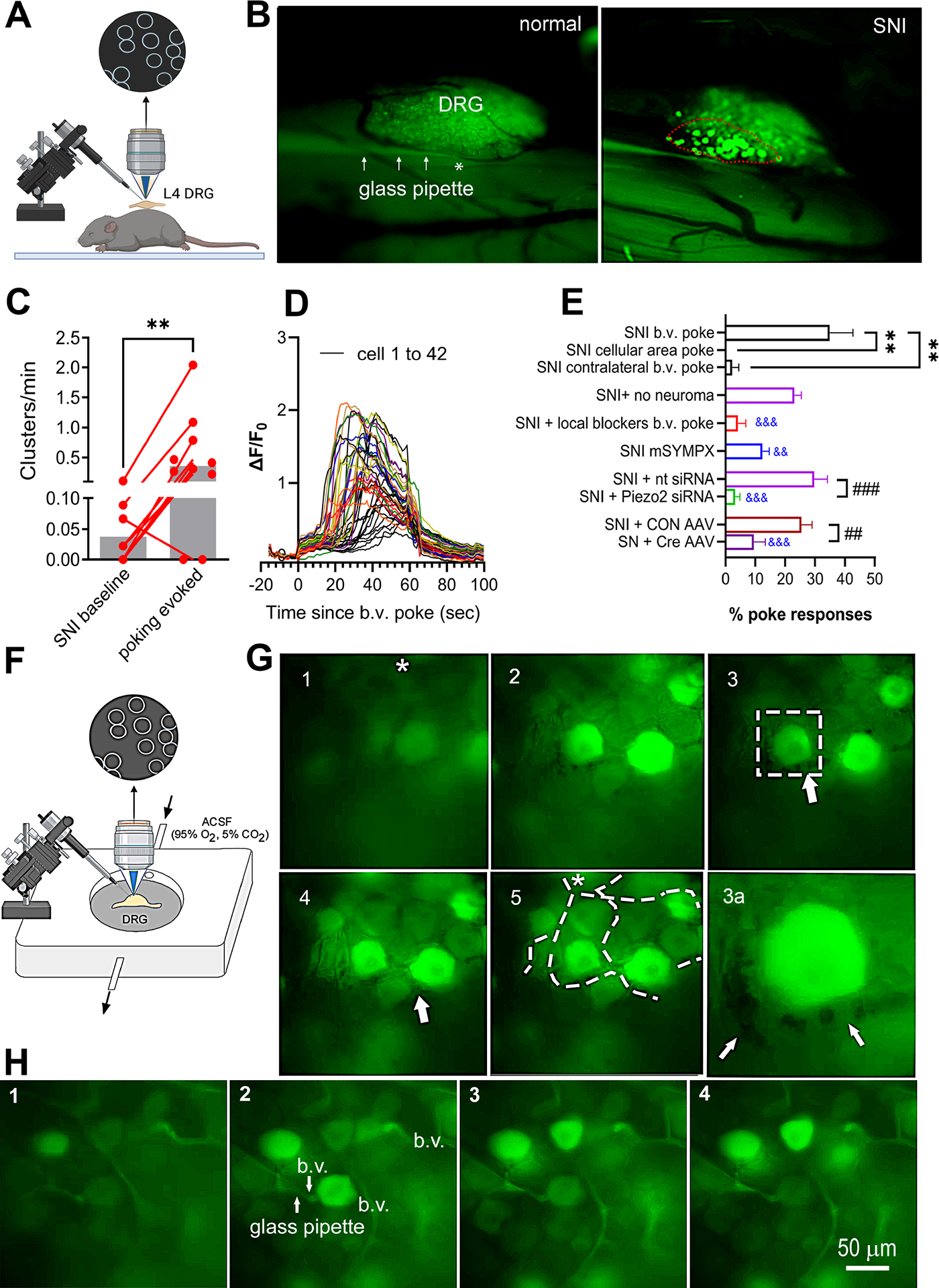
Clustered firing is evoked by mechanically induced myogenic vascular responses in the DRG from SNI mice in vivo and in vitro. (**A-E)** In vivo experiments. The L4 DRG was recorded in mice 3–5 weeks after SNI. After a 45-minute of baseline recording, a blunt glass micropipette was used to apply mechanical stimuli (~1 minute each) on visible blood vessels (A). (B) Sample images showing clustered firing triggered by gentle mechanical poking of the blood vessels in SNI DRG but not in normal, uninjured DRG. **(C)** Number of observed clusters normalized to the recording time (45 minutes for baseline, or total duration of mechanical stimuli). **, p<0.01, Wilcoxon matched pairs signed rank test. **(D)** Examples of calcium transients in response to poking a small blood vessel. **(E)** Summary data showing the percentage of blood vessel (b.v.) pokes that evoke clustered firing in the indicated conditions. & = significant difference when all experiments compared to SNI b.v. poke via ANOVA. * is for indicated comparisons (mixed effect model for the 3 groups with repeated measures in same animal), # unpaired t test for the 2 indicated comparisons. The poking-induced percent responses data in various experimental groups include data from animals used for the clustered firing data in Figures 2–4 (n=10). **(F-H)** Experiments with an *ex vivo* DRG preparation (n=5). **(F)** Diagram of the recording setup. Whole DRGs with nerve and root attached were removed from mice after SNI and mounted in a recording chamber perfused with ACSF at 35–36°. **(G)** Example still frames extracted from Movie S11 showing clustered firing evoked by gentle poking of a nearby blood vessel. Dashed lines showing the path of small blood vessels visualized under the microscope. The site of poking is indicated by *. 3a, the inset of frame #3 showing red blood cells moving in the capillary over the activated neuron. **(H)** Example still frames from Movie S12 showing the poking pipette, blood vessels visualized via i.v. injection of dextran, and activated neurons evoked by gentle poking.

To further test the vasomotion hypothesis, we used a fine glass pipette with blunt tip to apply gentle pressures locally to DRG blood vessels using a micromanipulator to evoke blood vessel movement via the myogenic responses (Figure 5A)^26^. We first confirmed that this poking stimulation triggered vasoconstriction and dilation, using vessels observed by in vivo recording in the periphery (Figure S5). We then recorded baseline clustered firing for 45 minutes in the ipsilateral L4 DRG of SNI mice, after which we mechanically stimulated several regions in turn in each DRG, including both larger blood vessels and cellular regions without vessels large enough to visualize with the low power objective lens used. Mechanically stimulating larger DRG blood vessels evoked clustered firing in 8 out of 10 SNI animals (Figure 5B–E, Movie S8). The poking-evoked clusters overall had similar characteristics to spontaneous and PE evoked clusters. The average number of neurons per poking-evoked cluster was 24.5±3.5, larger than the number of neurons/baseline cluster in the same animals (8.2±3.4), although similar to the number of PE-evoked cluster/neurons (29.9 ±9.3; Figure 2E–F). Some spatial overlap between spontaneous and poking-evoked clusters was observed in 5 out of 8 evoked clusters observed in animals that had spontaneous clusters. In 4 animals with no baseline clustered firing (45-minute baseline recording period), blood vessel poking could still evoke clustered firing. Two animals with baseline clustered firing failed to show poking-induced clustered firing. Overall, individual pokes activated clustered firing in 35% of the time.

Evoked clusters were generally near the poking site but could sometimes be more remote. When evoked by blood vessel poking, most clusters started firing within 5 to 20 seconds after the start of the mechanical stimulation (mean = 10.8 ± 1.9 seconds). Note that this was similar to the time course for poking-evoked blood vessel constriction in the periphery; we do not have an explanation for why this myogenic response is not faster. Normalizing the number of evoked clusters by total recording time to account for the very different times of observation (45 minutes for baseline, duration of ~1 minute per each mechanical stimulus for poking-evoked clusters) clearly demonstrated the much higher probability of evoking a cluster just after the mechanical stimuli (Figure 5C).

Poking blood vessels in the contralateral uninjured DRG only rarely evoked clustered firing (n = 1 cluster observed in 34 stimuli in 9 animals, same animals as in Figure 5E). Poking blood vessels at the same location in normal, uninjured control DRG (n=8) failed to evoke any neuronal responses in 8 mice tested (Figure 5B and Movie S9).

In animals that showed clustered firing evoked by blood vessel poking, poking in the cellular region away from larger blood vessels never evoked clustered firing (n = 31 pokes in 10 animals, same animals as Figure 5C), only single neuron firing (Movie S10). In a subset of SNI animals (n = 4) in which multiple poking stimuli were applied to various sites in the cellular area, 38.6 ± 3.1% of pokes evoked firing of a single cell. This was not significantly different from the rate of single cell firing observed in normal animals after cellular area pokes (33.6 ± 2.9%, n = 3, p = 0.45).

The clusters evoked by blood vessel poking shared many characteristics with spontaneous and vasoconstrictor evoked clusters (Figure 5E): they were still observed after the neuroma was disconnected, were blocked by intra-DRG application of D-GsMTx4 and gsmtx4, by siRNA against Piezo2 but not control siRNA injected i.t. 2 – 3 days prior to recording, and by AAV-Cre but not control AAV injection into mice with floxed Piezo 2. mSYMPX just prior to in vivo recording reduced the percentage of pokes that induced clustered firing, but a significant increase in the cluster frequency after poking could still be induced in animals with mSYMPX.

### Mechanically induced myogenic vascular responses could also evoke clustered firing in an isolated *ex vivo* DRG preparation

Our group has successfully used the *ex vivo* DRG preparation to record from individual neurons with microelectrodes, demonstrating that these neurons maintain their firing ability for several hours *ex vivo*^27^. We considered that blood vessel poking might still evoke clustered firing since this myogenic response depends on smooth muscle elements. In DRGs isolated from mice after SNI, clustered firing of neurons could be observed in response to blood vessel pokes, which sometimes also led to red blood cells in the capillaries moving near adjacent DRG neurons (Figure 5G–H and Movies S11–12). Poking- evoked clusters could be evoked in 6 out of 7 DRGs isolated after SNI, with an average cluster size of 10.5 neurons. In 8 normal DRG, no clusters could be evoked. For comparison, during in vivo recordings, poking-evoked clustered firing could be observed in 10 out of 12 animals, with an average cluster size of 21.1 neurons after SNI. In animals with mSYMPX prior to recording as also occurs with the *ex vivo* recording, 8 of 9 animals showed poking-evoked clustered firing with an average of 8.3 neurons per evoked cluster.

### Nerve injury increased the number of pericytes in the DRG and altered pericyte functionality

**Figure 6.**
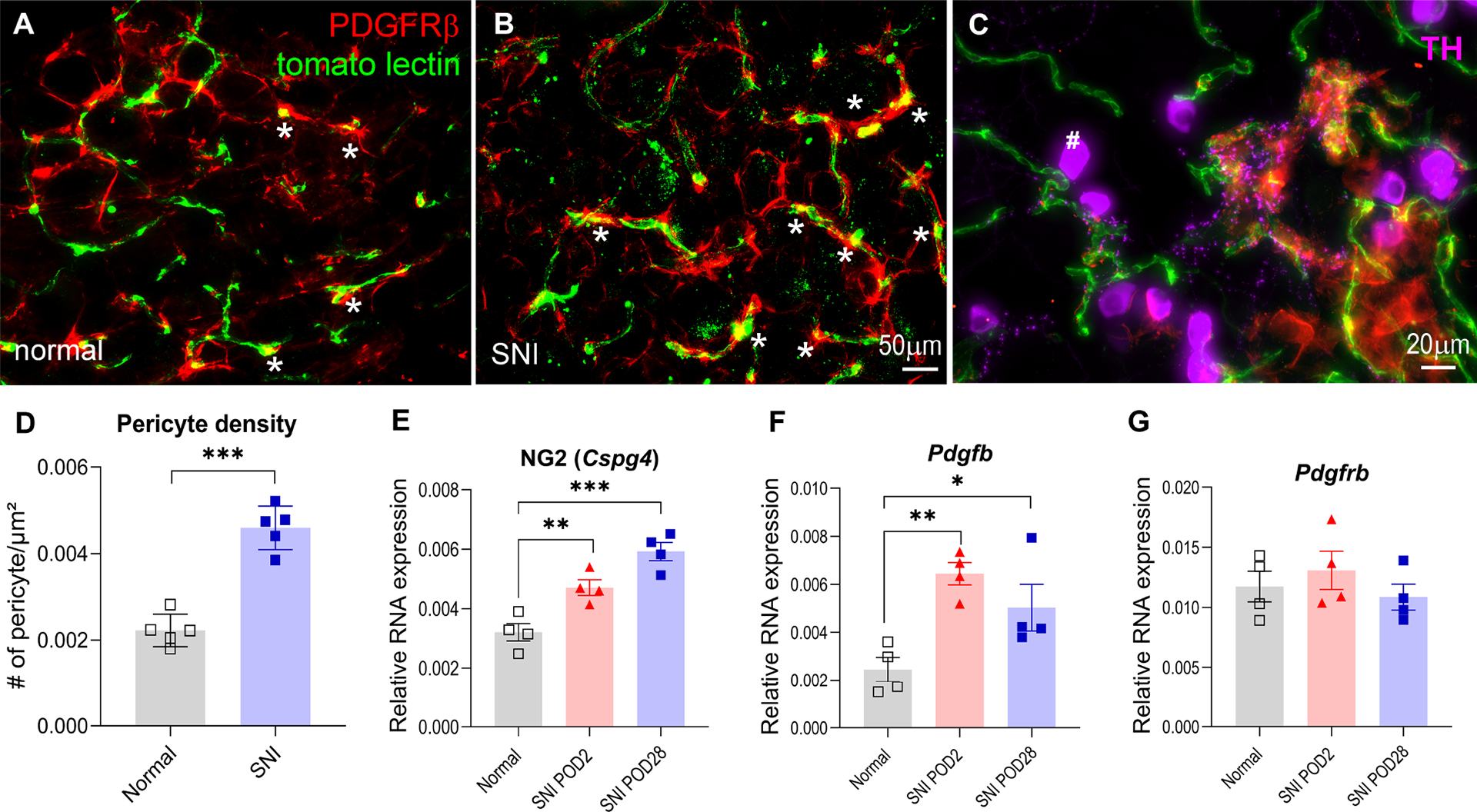
Peripheral nerve injury increased the density of pericytes in the DRG. **(A, B)** Representative image of pericytes identified by PDGFRβ (red) associated with tomato lectin labeled blood vessels (green) in normal and SNI mice on POD28. The asterisks indicate cell bodies (yellow) of the pericytes, where PDGFRβ signals overlap with tomato lectin-positive blood vessels. **(C)** Example of sympathetic (purple) innervations of the blood vessels (green) with pericytes surrounded (red) in SNI DRGs on POD28. #, TH-positive VGLUT3 sensory neurons. **(D)** Summary data showing the number of pericytes was higher in the SNI DRG compared to normal uninjured DRG (****, p <0.0001, t-test, n=5 per group). **(E)** qPCR measurements of NG2 expression showed it was increased in the SNI DRGs on POD2 and POD28. **, p<0.01; ***, p<0.001; n=4, one-way ANOVA. **(F)** qPCR measurements of Pdgfb mRNA expression showed it was increased in the SNI DRGs on POD2 and POD28. *, p<0.05; ***, p<0.01, one-way ANOVA; n=4. **(G)** qPCR measurements of Pdgfrb mRNA expression showed no change in the SNI DRGs on POD2 or POD28; n=4.

Activation of pericytes leads to vasoconstriction and altered blood flow in vivo^6,8^. We next sought to further elucidate the role of blood vessels in nerve injury-induced spontaneous pain and sensory neuron clustered firing, focusing on anatomical and functional changes of the pericytes in the DRG. On POD28, an increased density of pericytes in the DRG was observed (Figure 6A, 6B, 6D), suggesting pericyte proliferation in response to nerve injury. Pericytes were identified using PDGFRβ antibodies and tomato lectin was used for blood vessel visualization, with pericyte cell bodies appearing “yellow” to differentiate them from PDGFRβ+ neuronal signals (Figure 6A–B). Also, tyrosine hydroxylase (TH) positive sympathetic fibers were observed in some blood vessels with pericytes, suggesting interactions between sympathetic fibers and pericytes (Figure 6C, purple).

Increased pericyte density was confirmed by qPCR, which revealed elevated NG2 mRNA levels (Figure 6E), another pericyte marker. However, no significant changes were found in PDGFRβ mRNA, likely due to its expression in sensory neurons as well (Figure 6G). We also measured PDGFβ mRNA levels, the ligand for PDGFRβ, in the DRG, finding increased expression in SNI mice on POD 2, which persisted on POD28 compared to normal controls (Figure 6F). PDGFβ is crucial for pericyte recruitment and formation during blood vessel development^28^, suggesting its elevated levels likely contributed to angiogenesis and pericyte proliferation post-nerve injury.

**Figure 7.**
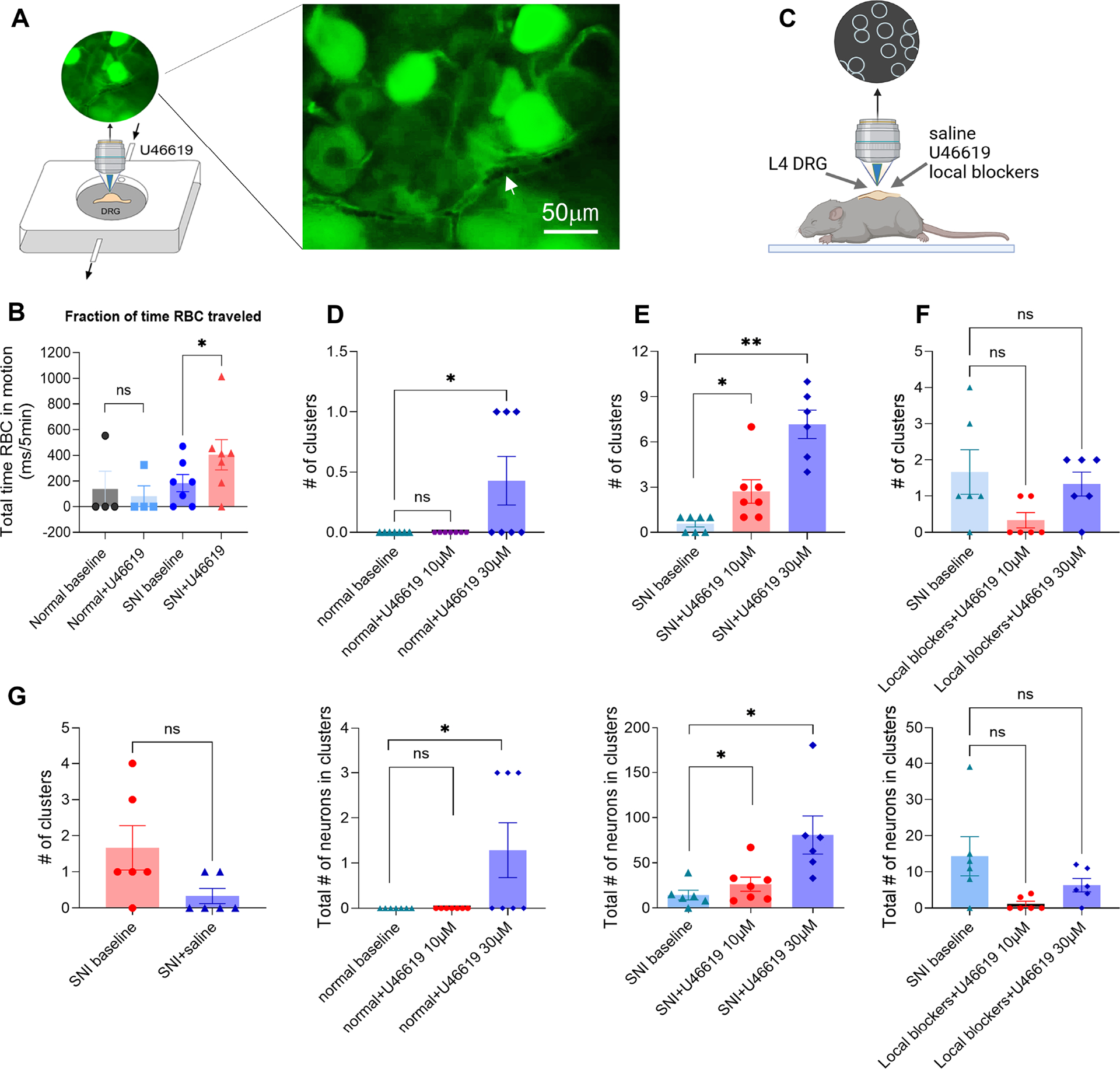
Pericyte activation alters the flow of red blood cells in vitro and triggers mechanically mediated clustered firing in vivo in SNI mice **(A)** Imaging setup used to measure red blood cells (RBC) movement in the *ex vivo* preparation. DRGs from normal or SNI pirt-GCaMP6s mice were dissected out and placed in a recording chamber continuously perfused with ACSF. Arrow indicates blood vessels containing RBCs. **(B)** Pericyte activator U46619 at 30 μM increased RBC movement in SNI but not in normal DRGs. *, p<0.05, n=9; paired t-test. **(C-E)** Pericyte activation triggers clustered firing in SNI mice on POD 28 in vivo. Baseline clustered firing was recorded for 45 min followed by topical application of U46619 (10 μM and 30 μM). Image recording continued for an additional 45 min. *p<0.05, **p<0.01, one-way ANOVA. D: Normal control DRG, n=7; E: SNI DRG, n=7. **(F)** Pericyte activator-induced clustered firing was blocked by co-application with mechanical channel blockers, D-GsMTx4 and gsmtx4. p>0.05, one-way ANOVA, n=6. **(G)** Topical application of saline did not evoke any clustered firing in 7 mice.

To assess the functional changes in blood vessel-associated pericytes after nerve injury, we conducted *ex vivo* whole DRG calcium imaging. On POD28, L4 DRGs from pirt-GCaMP6s mice were dissected out and placed in a recording chamber perfused with oxygenated artificial cerebrospinal fluid (ACSF) at 35–36°C (Figure 7A). Pericyte functionality was evaluated by measuring red blood cell (RBC) movement in small blood vessels, comparing SNI mice to normal controls. After establishing a baseline for 5 minutes, we added a pericyte activator, U46619 (30 μM in ACSF), and recorded RBC movement for an additional 10 minutes.

Pericyte function was quantified by calculating the fraction of time RBCs were in motion in the pre-identified blood vessels during 5-minute intervals. Results showed significantly increased and prolonged RBC movement in SNI mice compared to controls (Figure 7B). This suggests that SNI enhances pericyte activity, likely due to increased responsiveness to vasoactive stimuli, affecting microcirculatory dynamics within the DRG.

### Pericyte activation triggered clustered firing of sensory neurons in vivo

We were interested to know if this activation also triggered clustered firing in sensory neurons. We conducted in vivo calcium imaging of the L4 DRG in pirt-GCaMP6 normal control mice and SNI mice on POD28 (Figure 7C). After a 45-minute baseline recording, we applied buffered saline or U46619 at concentrations of 10 μM or 30 μM to the DRG, followed by a 10-minute incubation and an additional 45-minute recording.

U46619 at both concentrations induced clustered firing in SNI mice, resembling effects from PE and angiotensin II (Figure 7D–E, G). U46619 at 10 μM did not cause clustered firing in normal control mice, while 30 μM evoked a single clustered firing event in 3 of 7 normal mice, significantly less than in SNI mice.

To assess whether pericyte activation-induced clustered firings are mechanically mediated, we applied local mechanoreceptor blockers D-GsMTx4 and gsmtx4 before administering U46619. This application blocked baseline clustered firing and prevented U46619 (10 μM or 30 μM) from triggering new clustered firings (Figure 7F).

### Anti-VEGF monoclonal antibody blocked development of spontaneous pain and clustered firing

**Figure 8.**
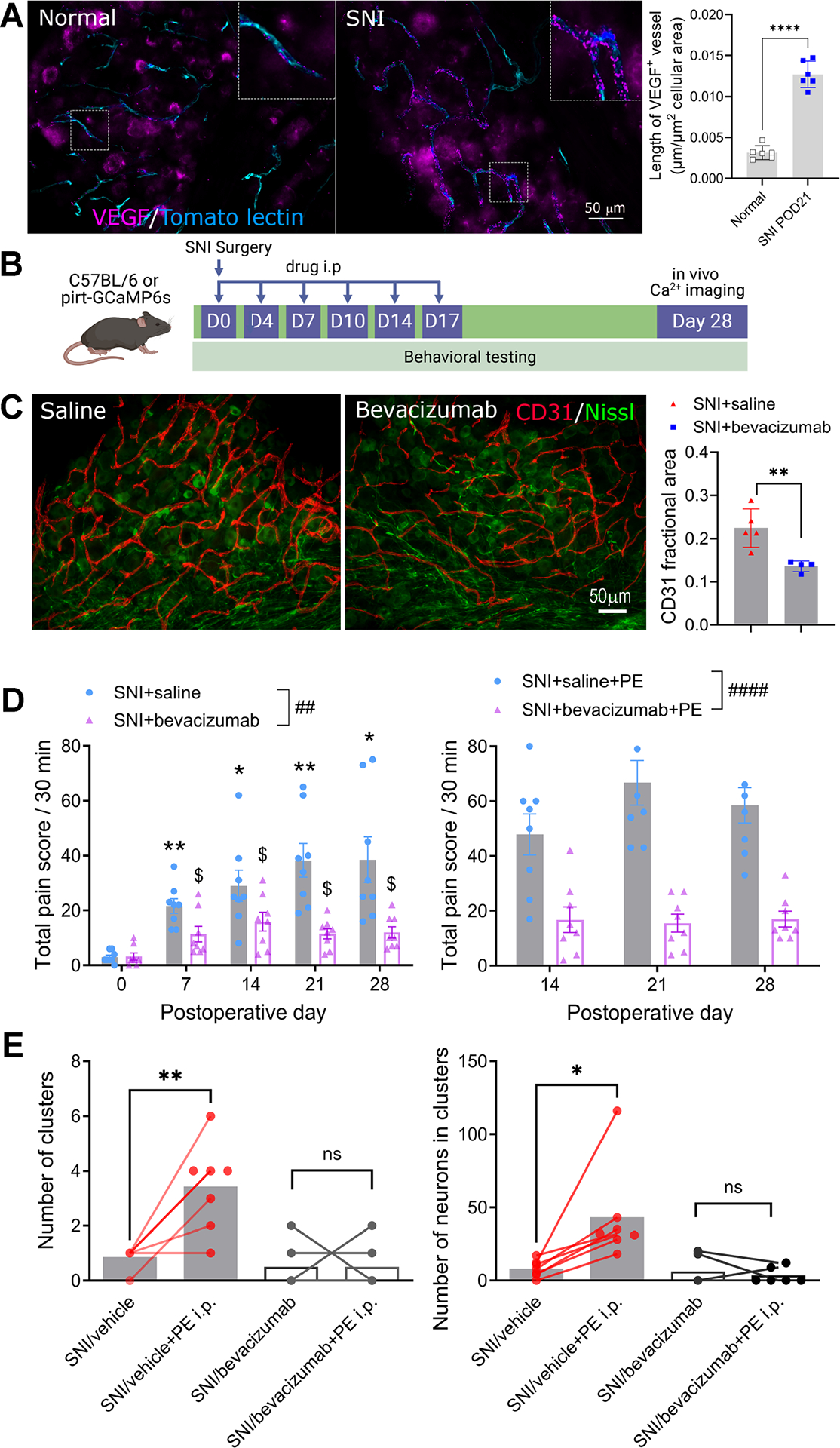
Anti-VEGF monoclonal antibody bevacizumab decreases spontaneous pain and blocks spontaneous and PE-evoked clustered firing. **(A)** Nerve injury increased VEGF protein expression along blood vessels measured by the length of VEGF-positive blood vessels normalized by cellular area. ****p<0.0001, unpaired t-test, n=6 per group. **(B)** Experimental strategy and timeline for C-E. **(C)** Bevacizumab administered during the first 3 weeks (10 mg/kg, i.p., twice a week) of nerve injury decreased CD31-postive blood vessel density. **(D)** Bevacizumab administered during the first 3 weeks of nerve injury decreased baseline (*, p<0.05, **, p<0.01; ##, p<0.01; $,p<0.05; two-way RM ANOVA with Šídák’s posttest; n=8 per group) and PE-evoked (####,p<0.0001, two-way RM ANOVA; n=8 per group) spontaneous pain scores. **(E)** Bevacizumab administered during the first 3 weeks of nerve injury blocked baseline (*, p<0.05, **, p<0.01, t-test, n=7 per group) and PE-evoked clustered firing (ns, not significant, t-test, n=6 per group).

To explore potential therapies for neuropathic spontaneous pain based on our findings, we tested the analgesic effects of bevacizumab, an FDA-approved anti-VEGF monoclonal antibody used in cancer treatment, which blocks angiogenesis^29,30^. Immunostaining of nerve-injured DRGs on POD21 showed an increased presence of VEGF protein in the blood vessels (Figure 8A).

Results indicated that bevacizumab (10mg/kg, i.p.) administered twice weekly for the first three weeks after nerve injury reduced blood vessel density (Figure 8B–C) and significantly lowered spontaneous pain scores beginning at week 1 post-surgery, with effects lasting over 4 weeks. Additionally, bevacizumab blocked PE-induced pain score increases observed on POD 14, 21, and 28 (Figure 8D). In vivo calcium imaging on POD28 also revealed significantly reduced clustered firing in the SNI mice treated with bevacizumab (Figure 8E). Although these findings are consistent with our vascular hypothesis, we cannot eliminate a possible additional contribution from direct excitatory effects of VEGF on sensory neurons^31^.

## Discussion

Our previous study^3^ demonstrated a correlation between spontaneous, intermittent firing of spatially clustered DRG neurons and spontaneous pain, that depended on the sympathetic nervous system. Our current study reveals a mechanism: bursts of spontaneous firing in sympathetic fibers, abnormally sprouting within the DRG after peripheral nerve injury, may lead to bouts of vasomotion (i.e. vasoconstriction, vasodilation and/or displacement due to local blood pressure changes) that activate nearby Piezo2-expressing neurons, resulting in the observed clustered firing and pain. This also explains why other vasoconstrictors, such as A-II, can similarly lead to clustered firing and spontaneous pain, as the blood vessel movement is “downstream” of sympathetic fiber firing. Consistent with this interpretation, acute local sympathectomy just prior to Ca^2+^ imaging did not eliminate the PE-evoked or poking-evoked clustered firing.

Our findings also provide one possible mechanism for the observed spatial clustering of spontaneously firing neurons, namely that they are all near a common region of vasculature and have injured axons. We observed PE-induced clustered firing only after spared nerve injury, not in normal DRG. Several factors may account for this observation, including hyperexcitability of the sensory neurons after SNI^32–34^, sprouting of sensory neurons to allow greater contact of Piezo2-expressing fibers with blood vessels^35–38^, increased/altered vascularization of the DRG after the peripheral nerve injury^4^, and activation of the hyper-reactive pericytes via α1 adrenoceptors^39,40^. This mechanism was proposed to account for pain induced by paw injections of vasoconstrictors in a rat model of complex regional pain syndrome or CRPS^41^, although in that study the hypersensitive site was in the periphery. We also observed blood vessel poking responses in an *ex vivo* preparation. Since blood flow is absent in this isolated preparation, and glucose and oxygen concentrations are maintained by the bath perfusion with oxygenated ACSF, this finding strongly suggests that the clustered firing in response to blood vessel constriction in vivo does not result from transient local hypoxia or low glucose concentrations.

It is highly unlikely that clustered firing in SNI DRGs induced by vasoconstrictors results from direct activation of the DRG neurons, because: 1) In SNI mice, up to two-thirds of the DRG neurons have injured axons and display hyperexcitability. If vasoconstrictors directly activated the sensory neurons, this would result in random firing of individual sensory neurons throughout the ganglion, rather than clustered firing; 2) The clustered firings triggered by vasoconstrictors were suppressed when mechanical receptors, including Piezo2 channels, were blocked pharmacologically or genetically.

The mechanism we have proposed here, where sensory neurons respond to mechanical signals from nearby blood vessels after nerve injury, is quite mechanistically distinct from the neurovascular coupling described in the CNS. In that case, activity in neurons (which largely lack Piezo2^42^) alters blood flow via communication between neurons, glia, and microvasculature cells^43^. While there are similarities between our findings in DRG neurons and migraine pain mechanisms, key differences exist. In migraine models, mechanical stimulation of sensory neurons by nearby vascular movement is thought to contribute to pain^44^. Sensitization or hyperexcitability of trigeminal neurons also plays a role, as observed in nerve injury models^45,46^. However, unlike in our study, the interaction in migraine occurs in the periphery with highly vascularized dura rather than within the sensory ganglion; the time course is much slower than the clustered firing observed in DRG neurons; and Piezo1 rather than Piezo2 is implicated. The relatively larger role of Piezo1 seems to be a difference between trigeminal and DRG neurons – expression of Piezo1 is much lower than Piezo2 in most studies of DRG neurons^47–49^ but is more robust in trigeminal neurons^44^.

The interpretation of our study is that sporadic clustered firing of DRG neurons conveys a signal to the spinal cord that is ultimately perceived as painful, as indicated by paw flinching. Our results are consistent with some previous studies implicating DRG Piezo2 in pain, especially evoked mechanical pain and allodynia. We now extend this role to account for spontaneous pain and place the locus of the interaction within the DRG.

We did not observe any clustered firing, spontaneous or vasomotion-evoked, in uninjured DRGs. Only neurons with injured axons contribute to clustered firing, likely due to hyperexcitability after nerve injury. Blood vessel movement in uninjured DRGs may not be sufficient to evoke neuronal firing via Piezo2 activation. Other factors contributing to spontaneous clustered firing may include nerve injury-induced angiogenesis and sensory fiber sprouting, which increase interactions between sensory neurons and blood vessels. Changes in the sympathetic nervous system, such as sprouting and dysfunction, may promote vasoconstriction via pericytes or endothelial cells, leading to clustered firing and spontaneous pain.

It is also possible that Piezo2 mediated, blood vessel-induced pain and clustered firing is caused by sudden changes in systemic or local (e.g., intraganglion) blood pressure. Elevated blood pressure or pulsation, from intrinsic factors or external stimuli like stress or physical activity, might increase mechanical pressure on injured sensory neurons, potentially leading to more frequent or intense clustered firing events and pain. This mechanism aligns with recent findings showing that pressure changes affect neuronal activity in the central nervous system^50^.

A limitation of the study is that only one measure of spontaneous pain, paw flinching behaviors, was used. While this seems most appropriate for the behavior likely to be elicited by sporadic bursts of cluster firing, improved measures of spontaneous pain would help the field move forward from its traditional focus on evoked reflexive pain behaviors.

In summary, our study presents compelling evidence for a causal link between vascular movements, clustered firing, and neuropathic spontaneous pain. Understanding the relationship between vascular dynamics and abnormal neuronal activity may provide new insights into pain mechanisms and pave the way for developing targeted therapies that address both the vascular and neural components of neuropathic pain in human patients.

## RESOURCE AVAILABILITY

### Lead contact

Further information and requests for resources and reagents should be directed to and will be fulfilled by the lead contact, Jun-Ming Zhang, MD, MSc, jun-ming.zhang@uc.edu

### Materials availability

This study did not generate new unique reagents.

### Data and code availability

- All data reported in this paper will be shared by the lead contact upon request.
- This paper does not report any original code.
- Any additional information required to reanalyze the data reported in this paper is available from the lead contact upon request.

## STAR Methods

### EXPERIMENTAL MODEL AND STUDY PARTCIPANT DETAILS

#### Ethic statements

The study was approved by the Institutional Animal Care and Use Committee (IACUC) of the University of Cincinnati, and the IACUC Review Boards at the Johns Hopkins University School of Medicine. Experiments were conducted in accordance with the National Institute of Health Guide for the Care and Use of Laboratory Animals.

#### Mouse strains

C57BL/6 mice were purchased from The Jackson (Jax) Laboratories (USA). Rosa26-lox-stop-lox GCaMP6s and Pirt-cre mice were obtained from the laboratory of Dr. Xinzhong Dong^51^. These two strains were crossed to express the calcium indicator GCaMP6s in sensory neurons but not sympathetic neurons^3^. Mice with Piezo2 flanked by loxP sites were obtained from Jackson Laboratories (strain 027720) and bred in-house.

Mice with initial age at 2–12 weeks-old from both sexes were used; data have been combined as no marked sex differences were observed. Sexes of mice for each figure are shown in Table S1. Mice were housed four per cage at 22 ± 0.5°C under a controlled 14/10 h light/dark cycle with free access to food and water. Animals were randomly assigned to experimental groups. The sample size of each experimental group was based on our previous similar studies^3,27^.

### METHOD details

#### Reagents

Phenylephrine and Angiotensin II were from R&D Systems. D-GsMTx4 and gsmtx4 were from Tocris/ R&D Systems. Phenylephrine stocks (10 mg/ml) and D-GsMTx4 or gsmtx4 stocks (250 μM) were made in buffered saline and stored at −20 C. Alexa 633 hydrazide (far red; label for elastin in arterial structures^52^, used for the whole mount image shown in Figure 1A, 1B, and tomato lectin Dylight 488 (50 μg/μl) used in Figure 5 were purchased from Thermo Fisher Scientific. Pericyte activator, U46619, stocks (1 μg/100 μl in DMSO) was purchased from Enzo Life Sciences and prepared in buffered saline for a final concentration of 10 μM and 30 μM for in vivo imaging and in ACSF for a final concentration of 30 μM for *ex vivo* whole DRG imaging.

#### siRNA injection

siRNAs directed against mouse Piezo2 mRNA (NCBI reference NM_001039485.4) were purchased from Dharmacon/Horizon and were a “smartpool” of 4 sequences: gaauguaauuggacagcga, ucaugaaggugcuggguaa, gauuauccauggagauuua, and gaagaaaggcaugagguaa; catalog number 667742. Sequence analysis showed that all four were predicted to target all of the Piezo2 splice variants described in^11^. The control non-targeting siRNA was catalog D-001210-02 (nontargeting control directed against firefly luciferase, screened to have minimal off-target effects and least 4 mismatches with all known human, mouse, and rat genes according to the manufacturer. 5 μL aliquots containing 133 pmoles of siRNA made up with cationic linear polyethylenimine (PEI)-based transfection reagent (“in vivo JetPEI”, Polyplus Transfection, distributed by VWR Scientific, USA) were injected intrathecally as follows: Two injections were performed, 6 hours apart, on one day. The i.t injection was applied in awake, conscious mice between the L4 and L5 level. A reflexive lateral flick of the tail was indicative of successful needle placement. The injection was done over the course of 3 seconds and following the injection the syringe was rotated slightly and removed. After the procedure, the mice were fully mobile and did not exhibit any signs of impairment^53^.

#### Virally mediated Piezo2 knockdown in DRG

As an alternative way to knock down sensory neuron Piezo2, mice with Piezo2 flanked by loxP sites were used. Cre recombinase under a neuronal promoter was delivered to the DRG by injecting it as a viral construct into the hindpaw. The construct pAAV-hSyn-Cre-P2A-dTomato was a gift from Rylan Larsen (Addgene viral prep # 107738-AAV9). The cre construct was injected mixed 1:1 with pAAV.CAG.GCaMP6s.WPRE.SV40 (AAV9) which was a gift from Douglas Kim & GENIE Project (Addgene viral prep # 100844-AAV9). We found that performing this injection around postnatal day 14 led to very efficient viral expression in the ipsilateral lumbar DRGs, that was largely but not completely confined to the DRGs innervating the paw. Control mice were injected with pAAV-hSyn-mCherry (AAV9) which was a gift from Karl Deisseroth (Addgene viral prep # 114472-AAV9), also combined with pAAV.CAG.GCaMP6s.WPRE.SV40 (AAV9). A red label was used to label infected neurons to avoid overlap with the GCaMP6s signal. Viral preps received from Addgene had titers in the range of 2 – 2.5 × 10^13^ GC/mL and were diluted 1:2 with PBS before use. The injection volume was 5 μl.

#### Spared nerve injury

The spared nerve injury (SNI) model was implemented as originally described in rat^54^ as modified to be used in mice^55^. Under isoflurane anesthesia, the 3 terminal branches of the sciatic nerve were exposed, and two branches (common peritoneal and tibial nerve) were tightly ligated with 8-0 silk suture, sparing the sural nerve. Then, the ligated branches were transected distal to the ligature, and around 2 mm of each distal nerve stump was cut. The incision was closed with sutures or tissue glue.

In some experiments a “microsympathectomy” (“mSYMPX”) was performed as originally described in rats^56^ and adapted for mice^27^. Briefly, the proximal L3 and L4 spinal nerves and transverse processes on one side were exposed. The spinal nerves (ventral rami) were visualized. The gray rami entering the L3 and L4 spinal nerves close to the DRG were transected by freeing the spinal nerve from surrounding tissues without exposure. Sham controls received similar exposure of the spinal nerves, but the gray rami were not cut. mSYMPX or sham surgery was performed just prior to (~15 minutes) in vivo recording in the indicated experiments. Successful mSYMPX following the above-described procedure was confirmed using previously described methods^27,57,58^ in 4 mice.

#### Behavior

Spontaneous pain was measured based on the methods described in our previous study^3^. Briefly, animals were placed in individual plexiglass containers and videotaped in the morning for 30 mins for 3 continuous days to allow acclimation to the chambers. Data from the 3^rd^ day was used for the spontaneous pain pre-drug baseline. In the afternoon of the same day, animals were injected with phenylephrine or vehicle, and behavior was taped for 60 minutes. Spontaneous pain was scored from the 15^th^ to the 45^th^ min to minimize any possible behaviors caused by injection-induced stress. The video recordings were subsequently played back and the number of bouts of licking and flinching/shaking/lifting with the ipsilateral hind paw was counted. Movements associated with exploratory behavior, locomotion, body repositioning, and grooming were excluded. One bout of licking was counted as 2 points, and one bout of flinching/shaking/lifting was counted as 1 point, and the total number of points for the 30-minute recording was used as the spontaneous pain score. The person scoring the behavior was blinded as to the experimental condition of the animal. The same procedures were used for Angiotensin II–induced spontaneous pain, except that the post injection data were obtained from the 30–60-minute period post-injection as we observed that this agent acted more slowly than phenylephrine.

#### Disconnection of neuroma

In some experiments, the connection between the neuroma and DRG was interrupted just prior to in vivo recording. The right side of sciatic nerve was exposed at the previous surgical site for SNI. The neuroma was located and carefully separated from the sural nerve, which was kept intact. The neuroma was then disconnected by cutting both common peroneal nerve and tibial nerve together at about 1mm proximal to the neuroma. The incision was closed in layers.

#### In vivo imaging and local drug application

Mice two months or older were anesthetized with isoflurane. A dorsal laminectomy over the DRG was performed, usually at spinal level L6 to S1 below the lumbar enlargement but without removing the dura. Bilateral L4 and L3 DRG transverse processes and spinous processes were removed so that DRGs on both sides could be imaged, with the contralateral DRGs serving as controls for some experiments. The exposed DRGs and spinal cord were rinsed with warmed (37°C) and oxygenated artificial cerebrospinal fluid (ACSF) to remove blood and covered with a thin layer of warmed (37°C) light mineral oil. The body temperature was maintained at 37 ± 0.5 °C on a heating pad and rectal temperature was monitored during exposure surgery and imaging. After exposure surgery, mice were laid in the abdomen-down position on the microscope stage where anesthesia was maintained with i.p. injection of sodium pentobarbital (40–50 mg/kg). Imaging was performed for 2 – 4 hours. The spinal column was stabilized using a pair of custom-designed glass hooks to minimize movements caused by breathing and heart beats. The microscope used was a fluorescence microscope (Olympus BX-UCB microscope system), which was equipped with a fast EM-CCD camera. Live images were acquired in timelapse mode at typically 1.25 to 1.67 Hz at depths below the dura ranging from 0 to 100 μm, using a 4x UPlanSApo dry objective at excitation 488 nm wavelength and emission at 500–549 nm for green fluorescence. At the end of each experiment, the viability of the DRG was confirmed by observing large numbers of cells giving a calcium transient in response to pinching of the hindpaw. In some experiments visibility of the blood vessels was enhanced by injecting high molecular weight dextrans (Texas Red^™^, 3000 MW, Lysine Fixable, ThermoFisher catalog D3328); this rendered blood vessels easier to see although they remained dark in the imaging setup.

For experiments examining local application of phenylephrine or U46619 to the DRG, part of the mineral oil covering the DRG was removed and 10 μl of phenylephrine, U46619, or the vehicle control (normal saline) was directly applied on top of the DRGs, without removing the dura mater. In experiments when bilateral DRGs were exposed, 50 μl of phenylephrine was added. For experiments examining local application of Piezo2 blockers, the blocker or its vehicle control (normal saline) was injected into the right L4 DRG. A very small hole was torn open in the dura mater between right L3 and L4 DRGs by using a pair of tweezers (size 5). A glass micropipette filled with 1–3 μl drug or control solution was carefully inserted into L4 DRG through the small hole and emptied very slowly (>1 min) by adding pressure through a 1 ml syringe, which was connected to the glass pipette via a 10 cm silicone tubing.

For experiments involving blood vessel or single cell poking, a small, polished glass pipette (diameter ~ 25 μm for in vivo recording, or 5 μm for *ex vivo* recording) attached to a micromanipulator was pressed against a visible blood vessel (or against single cells, as indicated). Stimuli had an average displacement of ~170 ± 14 μm, delivered over 5 seconds to evoke an all-or-none constriction of the blood vessel (“myogenic response”^26^).

As described in our previous study^3^, GCaMP6s was chosen as a highly sensitive calcium indicator with slow kinetics. Under our typical in vivo recording conditions (especially, lower magnification as used here), the dye gives a single integrated single waveform in response to action potentials separated by an interval on the order of 100–150 ms or less.

Raw images were collected and analyzed via Slidebook software. The contrast was adjusted and selected optical planes or z projections of sequential optical sections were used to obtain final images and to produce time-lapse movies.

#### *ex vivo* whole DRG imaging and mechanical poking

In some experiments, whole DRGs were removed from naïve or SNI mice and mounted into a chamber as previously used for our ex vivo whole DRG microelectrode recording^27^ and as originally described in rat^59^. The DRG and attached dorsal and ventral roots were removed from the animal. The DRG was secured in the recording chamber dorsal side up (i.e., with the same orientation as viewed during in vivo recording) and continuously perfused with ACSF (in mM: NaCl 130, KCl 3.5, NaH_2_PO_4_ 1.25, NaHCO_3_ 24, Dextrose 10, MgCl_2_ 1.2, CaCl_2_ 1.2, 16 HEPES, pH = 7.3) bubbled with 95% O_2_/5% CO_2_, maintained at 35 – 36°C. The microscope and imaging methods were as described above for in vivo recording, except that an Olympus LUMPlanFl 10x or 40x water immersion lens was used. Gentle poking of blood vessels was as for in vivo recording. Although there was no blood flow, vessels could still be visualized and contained some remaining red blood cells, which could be induced to make small back-and-forth movements after blood vessel poking. Image recording was limited to 30 minutes as the ex vivo clustered firing events could only be observed within this time frame. Direct quantitative comparison of *ex vivo* poking-evoked clusters with in vivo poking-evoked clusters was not attempted due to the differences in optics and sizes of glass filaments (~25 um for in vivo, ~5 μm for ex vivo), and because some of the blood vessels that were the hot spots for evoking poking-induced clusters in-vivo were not present in the ex vivo preparation due to removal of the dura.

#### *ex vivo* whole DRG imaging of red blood cell movements

Whole DRGs were harvested from naïve or SNI mice 15 minutes following intravascular injections of tomato lectin Dylight 488 (50 μg/50 μL). The DRGs were then placed in a recording chamber continuously perfused with oxygenated ACSF while keeping the temperature stable at 35–36°C. Vascular structures were visualized using tomato lectin. After confirming that the temperature in the recording chamber was stable, a region on the DRG surface that included one or more vessels containing red blood cells was selected and imaged for 5 minutes. Subsequently, the ACSF was replaced with U46619 solution (30 μM in ACSF) and imaging continued in the same area for another 10 minutes. The duration of red blood cell movements during both the 5-minute ACSF perfusion and the second 5-minute U46619 perfusion were measured and compared.

#### *in situ* hybridization

RNAscope was performed following the manufacturer’s instructions using the RNAscope Multiplex Fluorescent Reagent Kit v2 (Advanced Cell Diagnostics, Cat. No: 323110). The probes used in this study were mm-SCN10A (ACD, 426011, target range 165 – 1675 in NCBI reference NM_001205321.1) and mm-Piezo2 (ACD, 400191-C2, target range base pairs 983 – 1920 NCBI reference NM_001039485.4). The sequence range targeted by the Piezo2 probe was predicted to include all of the Piezo2 splice variants described in^11^. Images were captured using a Keyence BZ-X810 microscope, then images were analyzed by moving RGB TIF images into cellSens Imaging software.

#### Immunohistochemistry

Animals were first perfused with 0.1M phosphate buffer until clear fluid was seen, followed by perfusion with 4% paraformaldehyde for 20 minutes. For immunohistochemistry, neuroma, sciatic nerve, or DRG sections were cut at 30 μm on a cryostat after post fixation in 4% paraformaldehyde, 0.1M Phosphate Buffer, 4% sucrose. A no-primary antibody control was run to detect any non-specific binding of the secondary antibody. Images were captured using a Keyence BZ-X810 microscope or Olympus BX63 fluorescent microscope using Cellsens Dimension imaging software (Olympus), then images were analyzed by moving RGB TIF images into cellSens Imaging software.

For whole mount imaging of DRGs (Figure 1A, 1B), we adopted the clearing and staining methods^60^, with the addition of a methanol permeabilization step. Images were acquired with a Leica Stellaris 8 Confocal Microscope at the Live Microscopy Core at the University of Cincinnati College of Medicine.

The piezo 2 antibody was from Novus (catalog NBP1-78624, Rabbit polyclonal). It has been previously validated in a conditional knockout mouse^61^, and in studies using antibody-mediated affinity purification followed by mass spectrometry confirmation that the protein pulled down was Piezo2^62^. We also validated it by showing a large (73%) reduction in IHC signal in the DRG following i.t. injection of Piezo2 siRNA (Figure S3) and by observing decreased signal (to 2.82% of control value; p<0.0001) by preincubating the antibody with the immunizing protein. For staining blood vessels, the antibody used was CD31/PECAM-1 (Novus AF3628). In some experiments, an antibody to the regeneration marker GAP43 (Invitrogen PA5-34943) was used. This was validated in our previous study^63^. Nissl stain used to label neurons was from Neurotrace (TM 435/455 blue catalog N21479). PDGFRβ antibody used to label pericytes was from R&D Systems (Cat #: AF1042).

#### Quantitative PCR

Mice were deeply anaesthetized with pentobarbital sodium and transcardially perfused with cold PBS, then ipsilateral L4 DRGs were quickly dissected and frozen at −80°C. The RNA/Protein Purification Plus Kit (Norgen, Cat. #48200) was used to collect RNA 1–3 days later. Total RNA amount and quality were assessed using the SimpliNano UV-Vis Spectrophotometer (General Electric, USA) prior to conversion into cDNA using the High-capacity RNA-to-cDNA kit (Thermo Fisher, Cat. # 4387406). RT-qPCR with SYBR green master mix (ThermoFisher, Cat. #A25741) was performed using the QuantStudio 3 Real-Time PCR System (Thermo Fisher, Cat. # A28567). All samples were run in duplicate and target mRNA expression level was normalized to the average of Hprt and Gapdh mRNA. Relative expression of target genes was calculated based on the method described (Pfaffl MW, 2001). Primer sequences are in Table S2.

### QUANTIFICATION AND STATISTICAL ANALYSIS

Data were analyzed using GraphPad Prism version 9 or 10. Data are presented as mean ± S.E.M. or as scatterplots of individual data. In experiments with measurements from before and after a particular manipulation (e.g. drug application) lines are used to connect individual before-and-after points from each animal. The statistical tests used to evaluate the data are indicated in the figure legends. In graphs, the significance of differences between groups is indicated by the number of symbols, e.g. *, p<0.05; **, p<0.01; ***, p<0.001. Some diagrams were created with Bio-Render (schematics in all Figures).

## Supplementary Material

1 **Movie S1:** Frame-by-frame display of confocal images showing the blood vessels from a mouse DRG, related to Figure 1.
Green: all blood vessels labeled by CD31; Purple: arteries labeled by Alexa 633 dye.

2 **Movie S2**: Example of in vivo calcium image recording showing active neurons (GCaMP6, green) in clustered firings in a SNI mouse with intact neurons labeled (DiI, red), related to Figures 1 and 2.

3 **Movie S3**: Example of in vivo calcium image recording showing blood vessel movements preceding clustered firing events in a SNI mouse without PE, related to Figure 1I.
*Indicates location of the clustered firing and a blood vessel displaying dynamic changes (vasoconstriction and vasodilation).

4 **Movie S4:** Example of in vivo calcium image recording showing enhanced clustered firing events after PE administration (i.p.) in vivo in a SNI mouse, related to Figure 2.

5 **Movie S5**: Example of in vivo calcium image recording showing clustered firings overlap with 3 main branches of a blood vessel in a SNI mouse, related to Figures 1 and 2.
Also showing blood vessel movements preceding clustered firing events in vivo. *indicates clustered firing and blood vessel movement/displacement.

6 **Movie S6**: Example of in vivo calcium image recording showing clustered firing occurred after the onset of blood flow in an adjacent blood vessel from a SNI mouse related to Figure 1.

7 **Movie S7**: Example of in vivo calcium image recording showing clustered firing occurred after the displacement of an adjacent blood vessel from a SNI mouse, related to Figure 1.

8 **Movie S8:** Example of in vivo calcium image recording showing clustered firing evoked by gentle poking of a nearby blood vessel in a SNI mouse, related to Fig. 5.

9 **Movie S9**: Example of in vivo calcium image recording showing poking blood vessels or the cellular area of the DRG does not evoke any clustered firing in a mouse with intact nerve, related to Figure 5.

10 **Movie S10**: Example of in vivo calcium image recording showing poking blood vessel in the cellular area of the DRG in a SNI mouse does not evoke clustered firing, related to Figure 5.

11 **Movie S11:** Example of ex vivo calcium image recording showing clustered firing evoked by poking a small blood vessel, related to Figure 5.
Note: red blood cells can be seen rolling inside capillaries over the activated neurons in a SNI mouse.

12 **Movie S12**: Second example of ex vivo calcium image recording showing clustered firing evoked by poking a small blood vessel in a SNI mouse, related to Figure 5.

13

## Figures and Tables

**Table:** Key resources table

REAGENT or RESOURCE	SOURCE	IDENTIFIER
Antibodies		
Anti- Piezo 2 antibody	Novus Biologicals	Cat# NBP1-78624
Anti-CD31 antibody	Novus Biologicals	Cat# AF3628
Anti- GAP43 antibody	Invitrogen	Cat# PA5-34943
Anti-NeuN antibody	Sigma	Cat# N0142
Anti-GFP antibody	Novus Biologicals	Cat# NB100-1770
Anti-PDGFRβ antibody	R&D Systems	Cat #: AF1042
Donkey anti-Rabbit Secondary Antibody, Alexa Fluor 594	Invitrogen	Cat# A-21207
Donkey anti-Rabbit Secondary Antibody, Alexa Fluor 488	Invitrogen	Cat# A-21206
Donkey anti-Goat Secondary Antibody, Alexa Fluor 488	Jackson ImmunoResearch	Cat# 705-545-147
Donkey anti-Goat Secondary Antibody, Alexa Fluor 594	Jackson ImmunoResearch	Cat# 705-585-147
Bacterial and virus strains		
pAAV.CAG.GCaMP6s.WPRE.SV40	doi: 10.1038/nature12354.	Addgene Cat# 100844-AAV9
pAAV-hSyn-Cre-P2A-dTomato	Rylan Larsen Lab, unpublished	Addgene Cat# 107738-AAV9
pAAV-hSyn-mCherry	Karl Deisseroth Lab, unpublished	Addgene Cat# 114472-AAV9
Chemicals, peptides, and D-GsMTx4recombinant proteins		
(R)-(-)-Phenylephrine hydrochloride	Tocris	Cat# 2838 -100MG
D-GsMTx4	Tocris	Cat# 7170 -100μG
gsmtx4	Tocris	Cat# 4912 -100μG
Angiotensin II	R&D Systems	Cat# 1158 – 5MG
Alexa Fluor^™^ 633 Hydrazide	Thermo Fisher	Cat# A30634 -1MG
Dextran, Texas Red^™^, 70,000 MW, Lysine Fixable	Thermo Fisher	Cat# D1864-25MG
NeuroTrace^™^ 435/455 Blue Fluorescent Nissl Stain	Thermo Fisher	Cat# N21479 -1ml
FAST Dil^™^ oil; DilΔ9,12-C18(3), ClO4 (1,1’-Dilinoleyl-3,3,3’,3’-Tetramethylindocarbocyanine Perchlorate)	Thermo Fisher	Cat# D3899 -5 MG
Dextran, Fluorescein, 3000 MW, Anionic, Lysine Fixable	Thermo Fisher	Cat# D3306 -10MG
Triton X-100	Sigma	Cat# T9284-100ML
Paraformaldehyde Solution, 4% in PBS	Thermo Fisher	Cat# AAJ19943K2
Bovine Serum Albumin	Sigma	Cat# 9430-25G
U46619	Enzo Life Sciences	Cat# BML-PG023-0001
Critical commercial assays		
Total RNA Purification Plus Micro Kit	Norgen	Cat# 48200
High-Capacity RNA-to-cDNA^™^ Kit	Thermo Fisher	Cat# 4387406
SYBR green master mix	Thermo Fisher	Cat# A25741
RNAscope Multiplex Fluorescent Reagent Kit v2	Advanced Cell Diagnostics	Cat# 323110
RNAscope^™^ Probe- Mm-Scn10a	Advanced Cell Diagnostics	Cat# 426011
RNAscope^™^ Probe- Mm-Piezo2-C2	Advanced Cell Diagnostics	Cat# 400191-C2
in vivo-jetPEI^®^, in vivo nucleic acid delivery reagent	VWR	Cat# 89129-960
Deposited data		
Raw and analyzed data	This paper	doi:10.7945/b9hh-4595 https://doi.org/10.7945/b9hh-4595
Experimental models: Organisms/strains		
Mouse: C57BL/6 mice	Jackson Laboratories	Cat# JAX:000664 RRID:IMSR_JAX:000664
Mouse: *Pirt-Cre*	Xinzhong Dong Lab at Johns Hopkins University	N/A
Mouse: Rosa26-lox-stop-lox GCaMP6s	Xinzhong Dong Lab at Johns Hopkins University	N/A
Mouse: Ai96(RCL-GCaMP6s), *Rosa26-LSL-GCaMP6s*	Jackson Laboratories	Cat# JAX: 028866 RRID:IMSR_JAX:028866
Mouse: *B6(SJL)-Piezo2tm2.2Apat/J*	Jackson Laboratories	Cat# JAX: 027720 RRID:IMSR_JAX:027720
Oligonucleotides		
See Table S2 for a completed list of oligonucleotides		
Software and algorithms		
Slidebook 6	Intelligent Imaging Innovations, Inc.	N/A
Prism 9,10	GraphPad Software	N/A
Cellsens Dimension imaging software	Olympus	N/A
Other		
Confocal microscope	Leica	Stellaris 8
Fluorescent microscope	Olympus	BX63
Fluorescent microscope	Keyence	BZ-X810
QuantStudio^™^ 3 Real-Time PCR System,	Thermo Fisher	A28567
SimpliNano UV-Vis Spectrophotometer	General Electric	N/A

## HIGHLIGHTS

- Vasoconstrictors enhance cluster firing and neuropathic spontaneous pain
- Vasomotion-triggered cluster firings are mediated by Piezo2 of the DRG neurons
- Nerve injury increases the number of pericytes and angiogenesis in the DRG
- Anti-cancer, anti-VEGF monoclonal antibody blocks spontaneous pain and cluster firing
