# Supplementary material for: Vascular motion in the dorsal root ganglion sensed by Piezo2 in sensory neurons triggers episodic neuropathic pain
Source: Neuron. Author manuscript; Available in PMC 2025 Jun 30. (PMC12140901; doi:10.1016/j.neuron.2025.03.006)
Supplement: 13 [file NIHMS2063955-supplement-13.pdf]

Vascular motion in the dorsal root ganglion sensed by Piezo2 in sensory neurons triggers episodic neuropathic pain

Wenrui Xie[1], Debora Denardin Lückemeyer[1], Katherine A. Qualls[1], Arthur Silveira Prudente[1], Temugin Berta[1], Mingxia Gu[4], Judith A. Strong[1], Xinzhong Dong[2,3*], and Jun-Ming Zhang[1,5*]

[1] Pain Research Center, Department of Anesthesiology, University of Cincinnati College of Medicine, Cincinnati OH 45267, USA

[2] The Solomon H. Snyder Department of Neuroscience, Johns Hopkins University School of Medicine, Baltimore, MD 21209, USA

[3] Howard Hughes Medical Institute, Johns Hopkins University School of Medicine, Baltimore, MD 21209, USA

[4] Department of Anesthesiology & Perioperative Medicine, David Geffen School of Medicine, University of California, Los Angeles, Los Angeles, CA 90095, USA

[5] Lead Contact

[*] Corresponding authors. jun-ming.zhang@uc.edu (J.-M.Z. as lead contact) or xdong2@jhmi.edu

(XZ.D.)

SUPPLEMENTARY FIGURE LEGENDS, TABLES, AND MOVIES

Figure S1

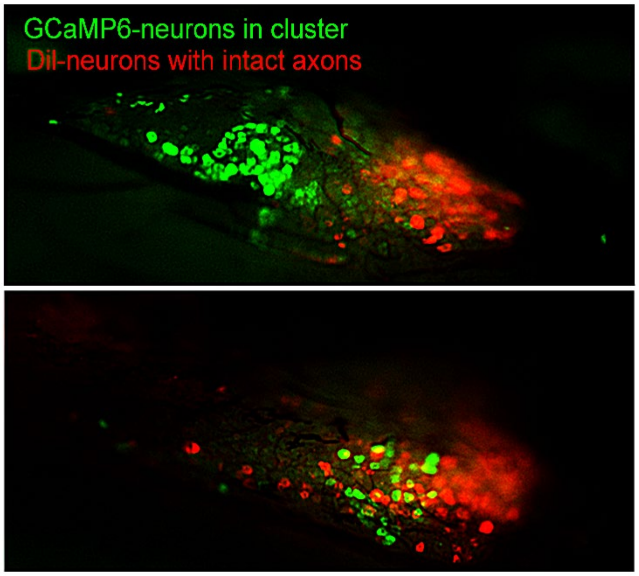

Figure S1: Intact uninjured neurons rarely contribute to clustered firing, related to Figures 1 and 2.

Still frame extracted from Movie S2 showing clustered firing events (green; GCaMP6) in animals in which the intact neurons were labeled by DiI injection into the paw after SNI surgery and 4-5 days prior to image recording. In 4 baseline clusters and a total of 111 neurons, only 3 of the neurons in the clusters were labeled by DiI. Top: an example of clustered firing neurons (green) occurred in a region outside of the intact uninjured neurons (red). Bottom: An example of clustered firing occurred in the region mixed with intact uninjured neurons. Note that very few intact neurons overlap with clustered firing neurons. n=7, 4 males and 3 females.

Figure S2

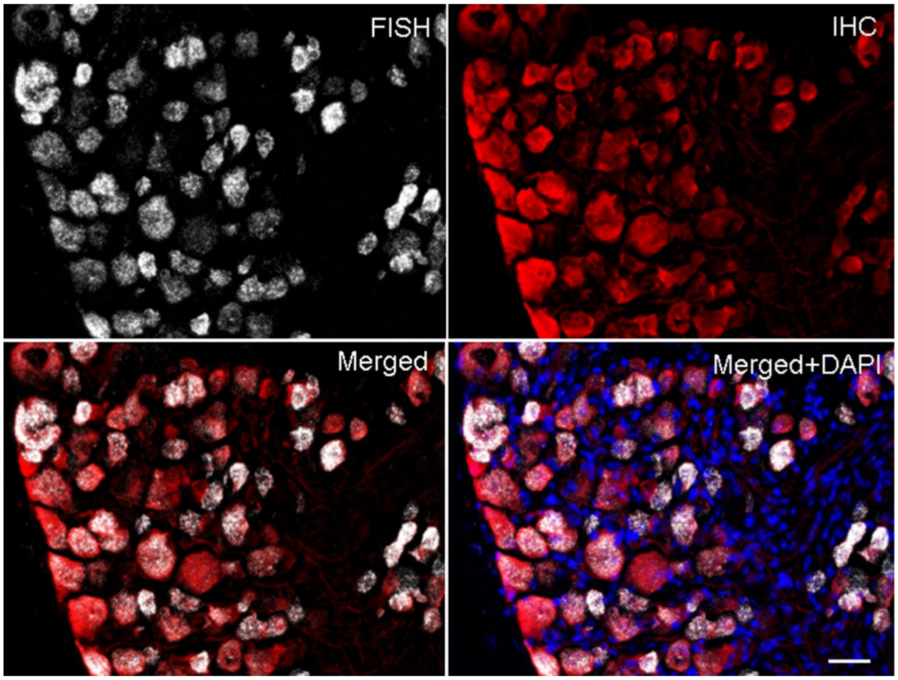

Figure S2: Piezo2 expression in mouse DRG, related to Figure 4.

In situ hybridization (RNA scope) and immunostaining of Piezo2 in normal DRG sections. Note Piezo2 is expressed in nearly all DRG neurons, with some neurons displaying higher expression levels as demonstrated in both methods. Scale bar=50 µm.

Figure S3

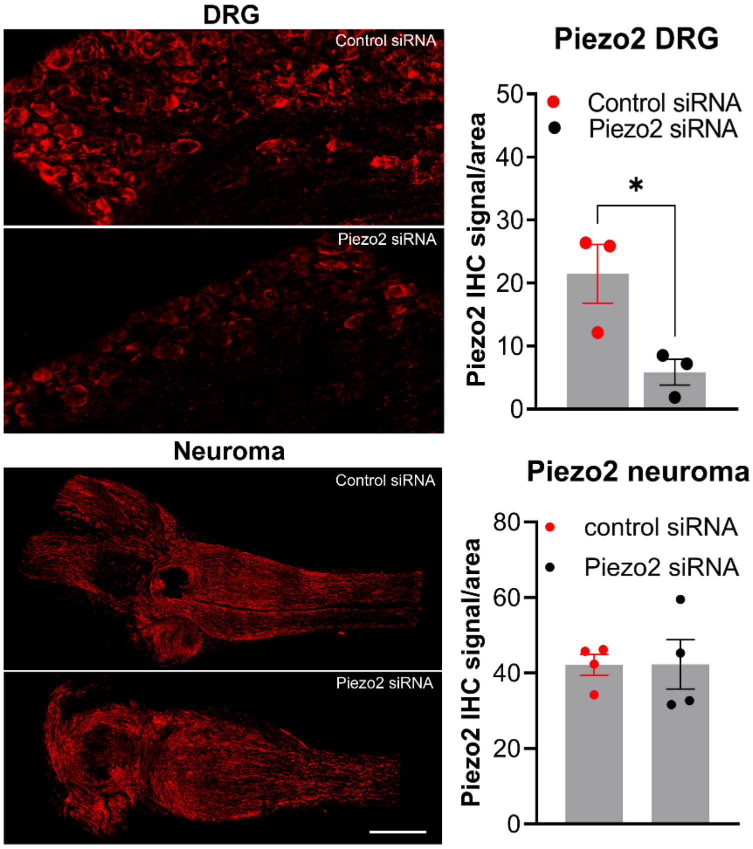

Figure S3: Knockdown of Piezo2 reduces Piezo2 expression in DRG but does not affect expression in the neuroma, related to Figure 4.

DRG sections were stained for Piezo2 in SNI mice, 2 days after injection of siRNA directed against Piezo2 or control siRNA. N = 3 male animals/group. Summary data: the density of Piezo2 labeling was significantly reduced by the Piezo2 siRNA, *, $p<0.05$, t-test. In separate experiments, the neuroma was isolated 30 days after spared nerve injury, and 2 days after i.t. injection of siRNA directed against Piezo2 or control nontargeting siRNA. The neuroma was sectioned (40 µm) and stained for Piezo2 (red). Bottom, example from Piezo2 siRNA-injected mouse, top, example from control siRNA-injected mouse. Summary data: the intensity of Piezo2 signal was not affected in the neuroma by Piezo2 knockdown regime ($p = 0.98$, t-test; n = 2 males and 2 females/group). Absolute values of intensity/area are in arbitrary units and are not directly comparable between the two experiments. Scale bar=200µm.

Figure S4

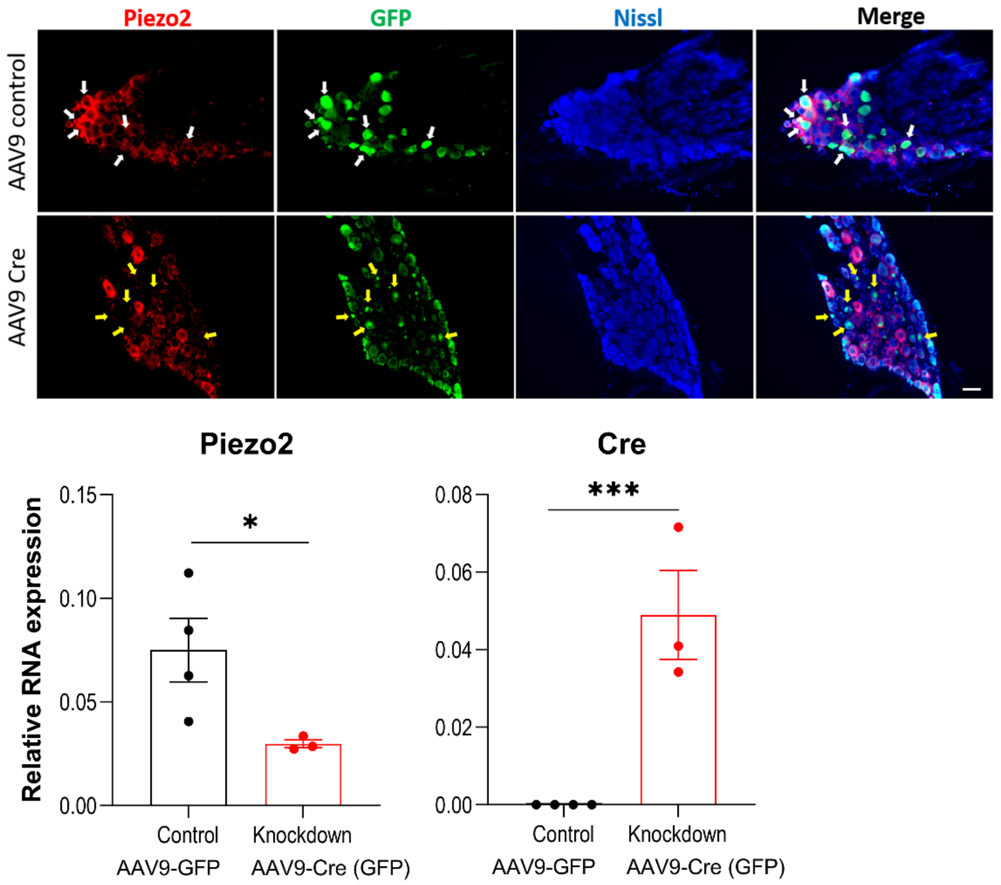

Figure S4: Confirmation of Piezo2 knockdown by AAV9-Cre injection into mice with floxed Piezo2 construct, related to Figure 4.

AAV9 expressing GFP and either Cre or control AAV9 expressing GFP alone was injected into the hindpaw during early life (see Methods). DRG were collected from adult mice and processed for immunohistochemistry or used for RNA isolation. Top: IHC of DRG labeled for GFP (green) Piezo2 (red) and neuronal label Nissl (blue). Arrows indicate examples of infected neurons co-expressing Piezo2 and GFP in control virus injected mouse, while Piezo2 is lacking in infected neurons co-expressing Cre. Bottom: qPCR measurements of Piezo2 expression showed it was reduced to 40% by Cre expression. *, $p=0.02$; ***, $p<0.001$, t-test, n=3-4 male mice/group). Expression of Cre mRNA was examined to confirm infection and correct genotyping. Scale bar=100 µm.

Figure S5

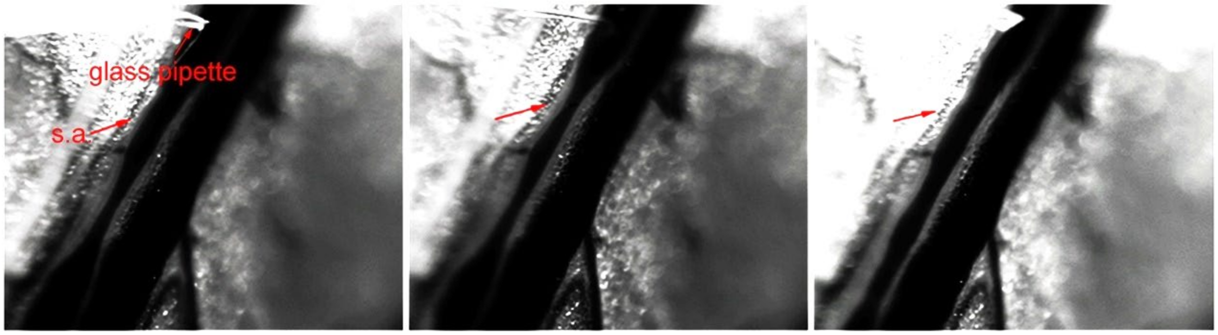

Figure S5: Myogenic responses of saphenous artery following gentle poking with a piece of blunt glass pipette, related to Figure 5.

s.a. saphenous artery; arrows indicate changes of the saphenous artery i.e. constriction/dilation.

Table S1

Figures	Male	Female
Figure 1E	3	3
Figure 1F	4	0
Figure 2A, uninjured mice, PE	4	4
Figure 2A, SNI, PE	11	10
Figure 2A, SNI, vehicle	7	4
Figure 2E, F, normal, i.p. PE	3	2
Figure 2E, F, normal, intraDRG PE	3	2
Figure 2E, F, SNI, i.p. PE	4	3
Figure 2E, F, SNI, intraDRG PE	5	2
Figure 2E, F, SNI, i.p. vehicle	5	3
Figure 2E, F, SNI, intraDRG vehicle	4	3
Figure 2E, F, SNI, mSYMPX, PE	4	5
Figure 2E, F, SNI, PE, neuroma removal	7	5
Figure 3A, normal, i.p. A-II	4	4
Figure 3A, SNI, i.p. A-II	6	6
Figure 3C, SNI, i.p. A-II	6	2
Figure 3C, SNI, i.p. A-II, D-GsMTx4 and gsmtx4	4	2
Figure 4B, SNI, D-GsMTx4 and gsmtx4, then PE	6	3
Figure 4B, SNI, D-GsMTx4 and gsmtx4, and PE	3	3
Figure 4D	3	2
Figure 4E	4	4
Figure 4F, control siRNA	4	3
Figure 4F, siRNA	4	4
Figure 4G, Cre group	8	5
Figure 4G, control AAV group	8	6
Figure 4H, Cre group	5	3
Figure 4H, RFP control group	4	3
Figure 5E	6	4
Figure 5F-H	3	2
Figure 6D	3	2
Figure 6E	4	0
Figure 6F	4	0
Figure 6F	4	0
Figure 7B	4	5
Figure 7C-E, normal group	4	3
Figure 7C-E, SNI group	5	2
Figure 7F	3	3
Figure 7G	4	3
Figure 8A	3	3
Figure 8D, baseline	4	4
Figure 8D, PE evoked	4	4
Figure 8D, baseline	4	3
Figure 8D, PE evoked	3	3

Table S1. Number of animals (male vs female) used in each experiment, related to Figures 1-8.

Table S2

REAGENT or RESOURCE	SOURCE	IDENTIFIER
Oligonucleotides		
Cre Forward – ATCTGGCATTTCTGGGGATTG	Invitrogen	NC_005856.1
Cre Reverse – GGCAACACCATTTTTTCTGACC	Invitrogen	NC_005856.1
Piezo2 Forward – GCACTCTACCTCAGGAAGACTG	Invitrogen	NM_001039485.4
Piezo2 Reverse – CAAAGCTGTGCCACCAGGTTCT	Invitrogen	NM_001039485.4
CD31 (*Pecam-1*) Forward – CCAAAGCCAGTAGCATCATGGTC	Invitrogen	NM_008816.3
CD31 (*Pecam-1*) Reverse – GGATGGTGAAGTTGGCTACAGG	Invitrogen	NM_008816.3
NG2 (Cspg4) Forward – GAGGTCTTGGTGAACTTCACCC	Invitrogen	NM_139001.2
NG2 (Cspg4) Reverse – GACAGTAGGAGACCGATGGTGT	Invitrogen	NM_139001.2
PDGFRβ (*Pdgfrb*) Forward – GTGGTCCTTACCGTCATCTCTC	Invitrogen	NM_001146268.1
PDGFRβ (*Pdgfrb*) Reverse – GTGGAGTCGTAAGGCAACTGCA	Invitrogen	NM_001146268.1
PDGFβ (*Pdgfb*) Forward – AATGCTGAGCGACCACTCCATC	Invitrogen	NM_011057.5
PDGFβ (*Pdgfb*) Reverse – TCGGGTCATGTTCAAGTCCAGC	Invitrogen	NM_011057.5
Hprt Forward – CTGGTGAAAAGGACCTCTCGAAG	Invitrogen	NM_013556.2
Hprt Reverse – CCAGTTTCACTAATGACACAAACG	Invitrogen	NM_013556.2
Gapdh Forward – AGGTCGGTGTGAACGGATTTG	Invitrogen	NM_001289726.2
Gapdh Reverse – GGGGTCGTTGATGGCAACA	Invitrogen	NM_001289726.2

Table S2. Oligonucleotides, related to STAR Methods.
